# Cultivating well-being in engineering graduate students through mindfulness training

**DOI:** 10.1371/journal.pone.0281994

**Published:** 2023-03-22

**Authors:** Wendy C. Crone, Pelin Kesebir, Beverly Hays, Shilagh A. Mirgain, Richard J. Davidson, Susan C. Hagness

**Affiliations:** 1 Department of Engineering Physics, University of Wisconsin-Madison, Madison, Wisconsin, United States of America; 2 Center for Healthy Minds, University of Wisconsin-Madison, Madison, Wisconsin, United States of America; 3 Healthy Minds Innovations, Madison, Wisconsin, United States of America; 4 Department of Orthopedics and Rehabilitation, University of Wisconsin-Madison, Madison, Wisconsin, United States of America; 5 Department of Psychology, University of Wisconsin-Madison, Madison, Wisconsin, United States of America; 6 Department of Electrical and Computer Engineering, University of Wisconsin-Madison, Madison, Wisconsin, United States of America; University of Valencia: Universitat de Valencia, SPAIN

## Abstract

The mental health crisis in graduate education combined with low treatment rates among engineering graduate students underscores the need for engineering graduate programs to provide effective methods to promote well-being. There is an extensive body of neuroscience research showing that contemplative practices, such as mindfulness, produce measurable effects on brain function and overall well-being. We hypothesized that a mindfulness-based training program designed for engineering graduate students would improve emotional well-being and, secondarily, enhance research capacity. An initial pilot study was conducted at a single institution (Phase 1), followed by a larger study conducted at both the original and a second institution (Phase 2) to gather additional data and show the program’s transferability. The program comprised eight weekly mindfulness training sessions. Individuals in the study were randomly assigned to either an intervention group or wait-list control group. We administered pre- and post-test surveys with quantitative measures designed to assess emotional and physical well-being, as well as creativity, research satisfaction, and desire to contribute to the betterment of society. Participants also completed a summative survey to evaluate the impact of the program on their well-being and research. Analysis revealed statistically significant findings: improved emotional health, decreased neuroticism, increased positive affect, decreased negative affect, and increased mindfulness in the intervention groups compared to the control groups. Intervention groups in Phase 2 also reported statistically significant improvement in satisfaction with their research. Our findings suggest that mindfulness training has the potential to play a vital professional and personal development role in graduate engineering education.

## Introduction

There is growing evidence of a mental health crisis in graduate student populations [1–4]. Depression and anxiety are experienced by graduate students at a rate that is six times that of the general population, and graduate students identify education-related issues as the most significant contributors [2]. A survey of graduate students at one U.S. institution showed that fields of study such as engineering and physical sciences reported depression at a rate of 43–46% [5].

One of the contributing factors is the level of stress graduate students experience and the lack of coping skills, resulting in adverse emotional, academic and health outcomes [6]. Graduate students face the pressures of coursework, qualifying/preliminary examinations, research, and publication in an environment that may be socially isolating and highly dependent on the quality of the professional relationship with an individual faculty advisor [7–9]. Simply the anticipation of a critical oral academic examination has been shown to negatively impact graduate students’ physical and psychological well-being by altering biological processes associated with stress response and immune function [10].

The impact of stress on the mental health is compounded by the trend of low treatment rates among engineering graduate students, with only a quarter of those with apparent mental health issues seeking help [11]. The stress experienced by graduate students who lack sufficient coping skills can exacerbate underlying mental health issues, negatively impact research progress and undermine degree completion [1, 12]. Calls for fundamental, systemic change in graduate education have been promulgated [13–15], but will take time. In the near-term, graduate students need effective methods to cope with the stress-inducing demands of their program.

We present an investigation of the impact of a mindfulness-based training program as a practical, cost-effective method for promoting well-being in the context of graduate engineering education. We recruited subjects to participate in an eight-week training designed to provide them with both an understanding of the scientific underpinnings of mindfulness practice and the experience of engaging with these practices firsthand. We hypothesized that training in mindfulness-based techniques would have a positive impact on graduate student well-being and their capacity to engage productively in research. We conducted survey-based assessments that were primarily quantitative in nature, augmented with qualitative data in the form of open-ended responses. Our investigation spanned multiple years involving training cohorts (each including an intervention group and a wait-list control group) at two research doctoral universities with very high research activity.

## Background

“Mindfulness” refers to a process of relating to the present-moment experience in an open, non-judgmental, curious, and accepting manner [16]. Mindfulness can be cultivated in a variety of meditation practices. A simple example is a sitting meditation in which the subject focuses on his or her own breathing, and repeatedly returns the attention to the breath without judgment when the mind wanders. Mindfulness meditation is a practice of mental training [17] aiming at “non-conceptual awareness” or development of a “beginner’s mind” [18]. The operating principle behind mindfulness meditation is that *state* mindfulness is heightened during the meditation practice, and over time this will in turn increase *trait* mindfulness, that is, one’s propensity toward mindfulness in everyday life [19].

There is an accumulating body of scientific evidence that mindfulness training can provide numerous benefits associated with mental health and general well-being. In clinical populations, mindfulness-based interventions have been found to be effective in improving mental health and alleviating suffering associated with physical, psychosomatic and psychiatric disorders such as anxiety, depression, eating disorders, and psychosis [20–24]. In non-clinical populations too, mindfulness training has been demonstrated to have a wide range of positive outcomes, including boosting attentional capacity, reducing stress, increasing emotional and physical well-being, strengthening the immune function, and promoting self-compassion, empathy, and perspective-taking [23, 25–30].

Prior studies have also explored the impact of mindfulness on a variety of student populations, and educators at the pre-kindergarten level through high school are introducing mindfulness-based practices as a means of improving student academic performance and emotional well-being [31]. Although mindfulness-based stress reduction programs have been shown to have efficacy within medical and psychology graduate student populations, e.g., [32–36], these types of interventions have only begun to be introduced to broader graduate student populations [37], despite extensive work that has been done at the undergraduate student level [38]. Our investigation contributes to this incomplete knowledge base specifically on effects of mindfulness training on the well-being of engineering graduate students.

## Methods

### Participants

Our investigation comprises two distinct phases of studies: Phase 1 was conducted over the course of one academic year at a single large public university (University of Wisconsin-Madison, denoted as University A) with one intervention group and one wait-list control group. Phase 2 was conducted over the course of multiple years at University A with a larger participant population (in comparison to Phase 1) and at a second large public university (University of Virginia, denoted as University B), with an intervention group and a wait-list control group in each of two cohort years (Year 1 and Year 2). University A is classified by the Carnegie Commission on Higher Education as a research doctoral university with very high research activity, located in the Midwest of the United States; University B is in the same Carnegie classification, but located in the Mid-Atlantic.

The human subject participation was approved and overseen by the Education and Social/Behavioral Science Institutional Review Board (IRB) at the University of Wisconsin-Madison (ID#2016–0917 for Phase 1 and ID#2018–1055 for Phase 2). All research was performed in accordance with relevant guidelines/regulations and informed consent was obtained from all participants. The IRB granted a waiver of signed consent so that the Research Participation Information and Consent Form could be obtained online.

**Fig 1:**
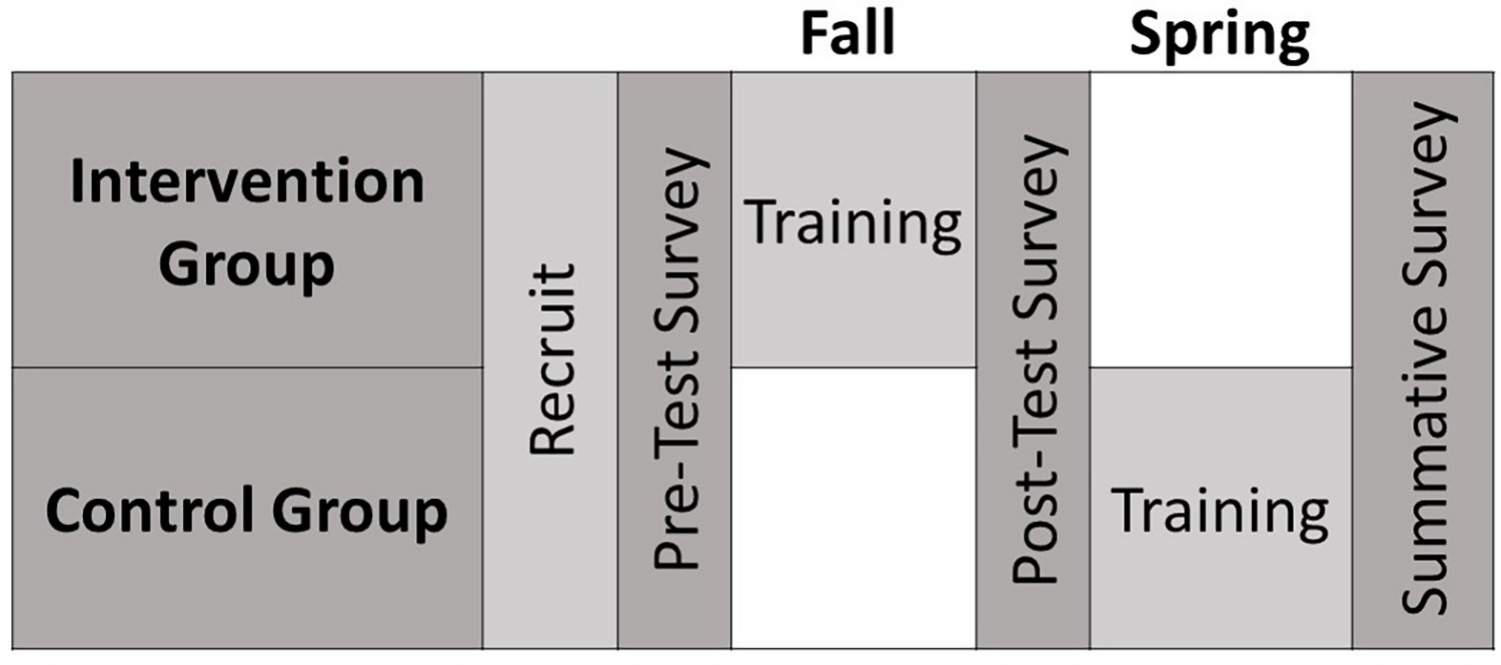
Timing of recruitment, training, and surveys for intervention and control groups. After recruiting participants and assigning them to either the Intervention Group or Control Group, all participants completed the Pre-Test Survey. After training of the Intervention Group all participants completed the Post-test Survey. The Summative Survey was completed by all participants after the Control Group completed training.

The Phase 1 participants were recruited from the general population of M.S. and Ph.D. graduate students in engineering departments at University A who identified themselves as being engaged in engineering research. People with a history of schizophrenia spectrum, bipolar disorders, or other psychotic disorders were excluded from participation. Participants were recruited through paper and electronic advertisements citing the general benefits of mindfulness training. The participants were randomly assigned to either an intervention group or a wait-list control group. This strategy enabled all participants to eventually receive the training while allowing for the control of many variables during data collection [39]. Participants in the intervention group completed the mindfulness training immediately (during Fall 2016) while participants in the control group completed it later (during Spring 2017). As illustrated in Fig 1, participants in both the intervention and control groups were given pre- and post-test surveys at the same time of the year (at the beginning and the end of the Fall semester), so that any difference observed between the two groups could be attributed to the effect of the mindfulness training and not differences in season or academic stressors that vary throughout the academic year.

Out of the 58 students who completed Phase 1 (i.e., who engaged in the intervention and completed both the pre-test and post-test surveys), 23 self-identified as female and 35 as male. They had a mean age of 27.12 (*SD* = 3.85). 33 participants self-identified as White, 14 as Asian, five as Hispanic, one as African American, and five as “Other”. 37 participants reported being born in the USA. Eight of the 58 graduate students were working toward a research-oriented M.S. degree and 50 toward a Ph.D. degree.

We recruited participants for Phase 2 from the general population of M.S. and Ph.D. graduate students in the engineering departments as well as computer sciences departments at University A and University B. Computer science students were added to the recruitment pool at University A in this phase to match the majors associated with both institutions. All participants identified themselves as being engaged in research. Recruitment, intervention/control group assignment, and all other major study aspects were conducted following the protocols and timing established for Phase 1. The one exception was that the Year 1 cohort participated in not only the summative survey as shown in Fig 1, but also a second summative survey (“final summative survey”) at the time at which the Year 2 cohort completed their summative survey. Year 1 training was offered in Fall 2018 (intervention) and Spring 2019 (control). Year 2 training was offered in Fall 2019 (intervention) and Spring 2020 (control). All pre-test and post-test surveys were completed in Fall 2018 (Year 1) or Fall 2019 (Year 2), prior to the onset of the COVID-19 pandemic in 2020. COVID-19 safety precautions interrupted the eight-week training for the Year 2 control participants after the fifth in-person class session. After a two-week delay, Year 2 control participants received their remaining three weeks of training remotely (online) after IRB approval of the delivery-mode modification. The Year 1 final summative and Year 2 summative surveys were conducted during the pandemic, after all cohorts completed the training.

Of the 157 participants who successfully completed Phase 2 (i.e., who engaged in the training and completed both the pre-test and post-test surveys and passed the attention checks), 73 self-identified as female, 83 as male, and 1 as transgender. They had a mean age of 27.53 (*SD* = 3.56). 81 participants self-identified as White, 53 as Asian, 11 as Hispanic, 5 as African American, and 7 as “Other”. 95 of the participants reported being born in the USA. 34 of the 157 graduate student participants were working toward a research-oriented M.S. degree and 123 toward a Ph.D. degree. S5 and S6 Tables provide participant and completion data for the two years of Phase 2.

### Training

The “Cultivating Transformative Research Through Mindfulness” (or *Mindful Engineer*, for short) curriculum was built on an existing “Cultivating Well-Being in the Workplace” curriculum developed by the Center for Healthy Minds and the UW-Madison School of Business Center for Professional and Executive Development. The “Cultivating Well-Being in the Workplace” program had been piloted regionally and nationally within a variety of companies and organizations, however it had not been empirically tested. The *Mindful Engineer* curriculum reported on here is a skill-based eight-week mindfulness training which draws from neuroscience-derived concepts detailed in *The Emotional Life of Your Brain* [40]. It comprises four components designed to cultivate well-being:

1. An introduction to and exploration of the six dimensions of emotional style–*Attention* (the ability to screen out distractions and stay focused), *Self-Awareness* (the ability to perceive one’s bodily signals that reflect emotions), *Resilience* (the ability to recover from negative emotion), *Outlook* (the ability to sustain positive emotion over time), *Social Intuition* (attunement to nonverbal social and emotional cues), and *Sensitivity to Context* (the degree with which one’s emotional and behavioral responses take into account the social context)–the combination of which characterize an individual’s specific overall emotional style;
2. An overview of the neuroplasticity of the brain and how the brain can be trained to change responses to emotions;
3. Training in mindfulness meditation and other contemplative practices, as well as cognitive skills and techniques; and
4. Strategies for creating and maintaining healthy mental and emotional habits.

The curriculum was developed in a culturally conscious way to be sensitive to the diverse and varied backgrounds and experiences of the graduate students, given the diversity represented at the institutions where the studies took place. The course content drew from the challenges of graduate school as a common denominator. The focus was to examine the underpinnings of how beliefs, opinions, biases, and behaviors are formed. Based on neuroscientific and psychological research, the participants were taught methods for developing insight into their biases, mental reactions, and habits in a non-judgmental manner. The cognitive and meditative tools provided throughout the course were designed to assist participants in managing emotional reactions and developing healthier perspectives, responses, and habits for improved resilience and emotional well-being.

The curriculum was delivered over a total of six semesters in Phase 1 and Phase 2, and in parallel at University A and University B during Phase 2. Instruction for both phases at University A was delivered by two instructors (BH and SAM) with over 20 years of personal meditation practice and over 10 years of meditation teaching experience. These instructors were also responsible for customizing the curriculum for this research investigation. Training for University B in Phase 2 was led by an instructor with over 20 years of personal meditation/mindfulness practice and 17 years of mindfulness/meditation instruction. This instructor received comprehensive in-person curriculum training and a detailed training manual in order to ensure content and delivery consistency across sites.

The curriculum consisted of eight weekly sessions of 50–60 minutes in duration. Our decision to offer eight instructor-led training sessions with limited expectations for practice outside of the formal sessions aligns with the recommendations of a recent meta-analysis [41] of 25 studies involving mindfulness-based interventions for undergraduate and postbaccalaureate students. Each weekly session focused on a specific emotional style building on the previous weeks’ content (see S1 Table for an outline). Practice teams were established at the beginning of the training and maintained throughout its duration to act as a support community for participants and to assist in fostering continuing dialogue and reflection between sessions.

The training provided numerous opportunities for cognitive and contemplative skill-building in and outside the “classroom.” Each session began with a ten-minute check-in for sharing, discussion, and answering questions regarding the previous week’s home practice experience. In addition, time was allotted for reflection and discussion following each new meditation and cognitive exercise. Time spent on meditation and cognitive exercises, including reflection and discussion, was between 20–25 minutes per class session. The remainder of the time was spent introducing the emotional style being discussed and related concepts and content. The course emphasis was not only on learning the meditation techniques, but also on helping participants build insights, skills and strategies to support integrating mindfulness practices into their daily lives.

Attention and self-awareness were considered key foundational concepts in developing a mindfulness practice and were covered in greater depth–two sessions each. Sessions 1 and 2 were devoted to the science of attention and the benefits of strengthening mental focus using techniques such as breath-focused meditations. Sessions 3 and 4 were devoted to the concept of meta-awareness and cultivating self-awareness of the mind-body connection as experienced through a body scan meditation. Session 5 (resilience) focused on the impact of stress on well-being and personal effectiveness, and cognitive reframing practices to enhance resilience in stressful times. Participants were also introduced to an open awareness meditation for maintaining mindfulness and staying present in challenging situations. Session 6 (positive outlook) focused on exploring the concepts of balance and equanimity, seeing and savoring positive experiences, practicing gratitude daily, and learning a meditation practice to open one to seeing potential benefits in challenging situations. Session 7 (social intuition) focused on cultivating the ability to read social cues, exploring connection as a basic human need, recognizing personal biases and deterrents to connecting, and practicing exercises in reading social cues and mindful listening. Session 8 (sensitivity to context) focused on recognizing and regulating emotions in a context-sensitive fashion, learning to stabilize reactions through balanced breath exercise, and using loving-kindness meditation to strengthen compassion and non-judgmental acceptance of self and others.

In addition to the guided meditations and exercises conducted in the weekly sessions, participants were encouraged to practice formal mindfulness meditation (e.g., breath meditation, body scan meditation) daily for at least 10 minutes per day and to integrate brief mindfulness practices daily through the duration of the study. Each week the participants were reminded of the guided meditations and exercises they had learned so far and were asked to focus some of their weekly practice time on recently acquired skills. Additionally, on-line resources, including audio recordings of all meditations as taught during the course, were provided weekly to support participants in developing their practice and incorporating mindfulness into their daily life. Open-ended responses to the question “How much time did you devote to mindfulness activities in an average week?”, which was asked of the intervention group in the post-test immediately following the completion of training, indicated that the average practice time was approximately an hour-and-a-quarter per week in Phase 1 and approximately an hour per week in Phase 2. This was consistent with the practice time requested of the participants outside the “classroom.”

### Measures

Pre-test, post-test, and summative surveys were administered following standard practices of the University of Wisconsin-Madison and with approval of the IRB of the University of Virginia. Participants completed all the measures on the online survey platform Qualtrics. We employed a variety of well-being-related measures, as well as mindfulness-related measures and custom measures related to research satisfaction. These are described in detail below.

### Emotional Style Questionnaire (ESQ)

We used the 48-item ESQ to investigate the changes in participants’ emotional lives as a result of the training. This was a revised version of the questionnaire found in *The Emotional Life of Your Brain* [40] and an earlier version of the empirically validated 24-item ESQ [42]. Participants indicated, on a scale ranging from 1 (*strongly disagree*) to 7 (*strongly agree*), how much they agreed with several statements regarding their emotional patterns. There were eight items for each of the six Emotional Style dimensions covered in the training curriculum. The overall scale had good internal reliability (Cronbach’s alpha = 0.86 at pre-test and .85 at post-test; note that all Cronbach’s alpha values are reported for Phase 1 to establish the reliability of the measures.). Sample scale items include: “When bad things happen, I am usually good at seeing the positive in them” (*Outlook*); “When I experience a setback, I do not stay demoralized for too long” (*Resilience*); “I am good at reading facial expressions and body language” (*Social Intuition*); “I am usually clear about what I’m experiencing emotionally and rarely confused about my emotions” (*Self-Awareness*); “I have occasionally been told that I behaved in a socially inappropriate way” (*Sensitivity to Context*), and “I have good concentration skills” (*Attention*). The alphas for the dimensions at pre-test and post-test were as follows, respectively: Outlook (.75; .78), Resilience (.80; .70), Social Intuition (.74; .82), Self-Awareness (.61; .68), Sensitivity to Context (.81; .82), and Attention (.86; .88). We calculated participant scores for each of the six dimensions, as well as the overall scale score (i.e., the average of all 48 items). This score serves as an indicator of general emotional health and is known to correlate with other physical and mental well-being measures.

### Ten Item Personality Inventory (TIPI)

To capture participants’ personality, we used the TIPI, which has been validated as a brief measure of Big Five personality [43]. On a scale ranging from 1 (*strongly disagree*) to 7 (*strongly agree*), participants rated the extent to which 10 pairs of personality traits apply to them, such as “extraverted, enthusiastic” (Extraversion), “anxious, easily upset” (Neuroticism), “sympathetic, warm” (Agreeableness), “dependable, self-disciplined” (Conscientiousness), and “open to new experiences, complex” (Openness to Experience).

### Positive and Negative Affect Schedule (PANAS)

Participants indicated to what extent they have been experiencing certain negative and positive emotions “during the past two weeks including today.” The scale consisted of 10 positive (e.g., attentive, enthusiastic) and 10 negative emotion words (e.g., upset, guilty). Responses could range from 1 (*very slightly or not at all*) to 5 (*extremely*) [44]. For Positive Affect, Cronbach’s alpha was .91 at pre-test and .90 at post-test. For Negative Affect, it was .87 at pre-test and .90 at post-test.

#### Cohen-Hoberman Inventory of Physical Symptoms (CHIPS)

CHIPS is a list of 39 common physical symptoms (e.g., back pain, cold or cough, acne, nosebleed) [45]. Participants were given this list and asked to put a check mark next to each symptom that has bothered or distressed them “during the past two weeks including today.” This served as a measure of physical well-being.

#### Mindful Attention and Awareness Scale (MAAS)

On a scale ranging from 1 (*almost never*) to 6 (*almost always*), participants responded to 15 items capturing awareness of and attention to what is taking place in the present [46]. Sample scale items include “I do jobs or tasks automatically, without being aware of what I’m doing” and “I find it difficult to stay focused on what’s happening in the present.” (Cronbach’s alpha = .83 at pre-test and .84 at post-test).

#### Five Facet Mindfulness Questionnaire—Short Form (FFMQ-SF)

We employed the 24-item short version of the FFMQ to assess different aspects of mindfulness [47]. The FFMQ-SF has 5 subscales: Observe (e.g., “I pay attention to physical experiences, such as the wind in my hair or sun on my face), Describe (e.g., “I’m good at finding the words to describe my feelings”), Act with Awareness (e.g., “I find myself doing things without paying attention”), Non-judge (e.g., “I think some of my emotions are bad or inappropriate and I shouldn’t feel them”), and Non-react (e.g., “I watch my feelings without getting carried away by them”). Participants indicated their agreement with these statements on a scale from 1 (*strongly disagree*) to 7 (*strongly agree*). Cronbach’s alpha for the whole scale was .83 at both pre-test and post-test. The alphas for the subscales at pre-test and post-test were as follows, respectively: Observe (.74; .73), Describe (.85; .81), Act with Awareness (.87; .92), Non-judge (.77; .84), and Non-react (.85; .85).

#### Research Satisfaction Scale

We developed eight face-valid items to measure participants’ satisfaction with their research and their ability to make progress in their research. These items tapped into participant perceptions of making progress (e.g., “I am satisfied with how my research is progressing”), ability to do creative research (e.g., “I feel creative and innovative in my research”), and ability to persist in the face of obstacles (e.g., “I feel like I’m able to overcome obstacles in my research”). Participants indicated their agreement with these statements on a scale from 1 (*strongly disagree*) to 7 (*strongly agree*). The scale yielded acceptable internal reliability (Cronbach’s alpha = .86 at pre-test and .90 at post-test). The complete scale is provided in S1 Methods.

#### Contributive desire scale

We developed five statements to evaluate participants’ desire to contribute to the well-being of other people and the betterment of society, particularly through conducting impactful research. Scale items included “I am motivated to use my knowledge and skills to make a difference in people’s lives” and “I feel a responsibility to do things to improve the well-being of people.” The complete list of items is provided in S1 Methods. Participants indicated their agreement with these statements on a scale ranging from 1 (*strongly disagree*) to 7 (*strongly agree*). Cronbach’s alpha for the scale was .87 at pre-test and .81 at post-test.

#### Summative and Final Summative Surveys

The summative survey included open-ended questions regarding the impact and value of the training related to participants’ professional work and personal life. Questions included “In what ways has the training impacted your research and other professional work?”, “In what ways has the training impacted your personal life?”, and “What was most valuable to you about the training?”. Participants were also asked to report on the amount of time, frequency, and type of mindfulness activities that they were currently doing. All participants completed the Summative Survey at the end of the study year (within 12 months of the completion of their training). Phase 2 Year 1 participants were asked to complete the assessment again as a Final Summative Survey 18–24 months after the completion of their training.

## Results

### Phase 1: Quantitative measures

The data described below comes from participants who completed at least 75% of the training. Some attrition occurred prior to the start of and over the course of the study, and not all participants completed the eight-week training. Participants who did not attend the training or had poor attendance chose not to complete the survey instruments. The initially enrolled sample was 73 (38 were assigned to the intervention group and 35 to the control group), composed of individuals who consented to the study and completed the pre-test. 24 participants in the intervention group and 34 participants in the control group completed both the pre- and post-test surveys and passed all built-in attention checks. The summative survey, conducted after both the intervention and control groups had completed the training, was completed by 14 participants assigned to the intervention group and 21 to the control group.

The majority of the participants (48 out of 57 respondents) had little to some familiarity with mindfulness prior to the study, as indicated by their responses to a question in the pre-test. Six reported no familiarity at all and three reported “a great deal” of familiarity.

Independent t-tests were conducted to compare groups at baseline (i.e., at pre-test) for demographic variables and outcome measures. Despite the use of random assignment to groups, analyses revealed that those assigned to the intervention group were significantly lower on the *Resilience* subscale of the ESQ (*p* = 0.007) and higher on the *Neuroticism* subscale of the Ten Item Personality Inventory (*p* = 0.019) at pre-test than the control group. There were no significant differences for any of the other variables (*p*’s > 0.05). Importantly, we controlled for any differences in cohort characteristics in the analysis by analyzing the impact of the training relative to baseline, rather than absolute impact.

**Fig 2:**
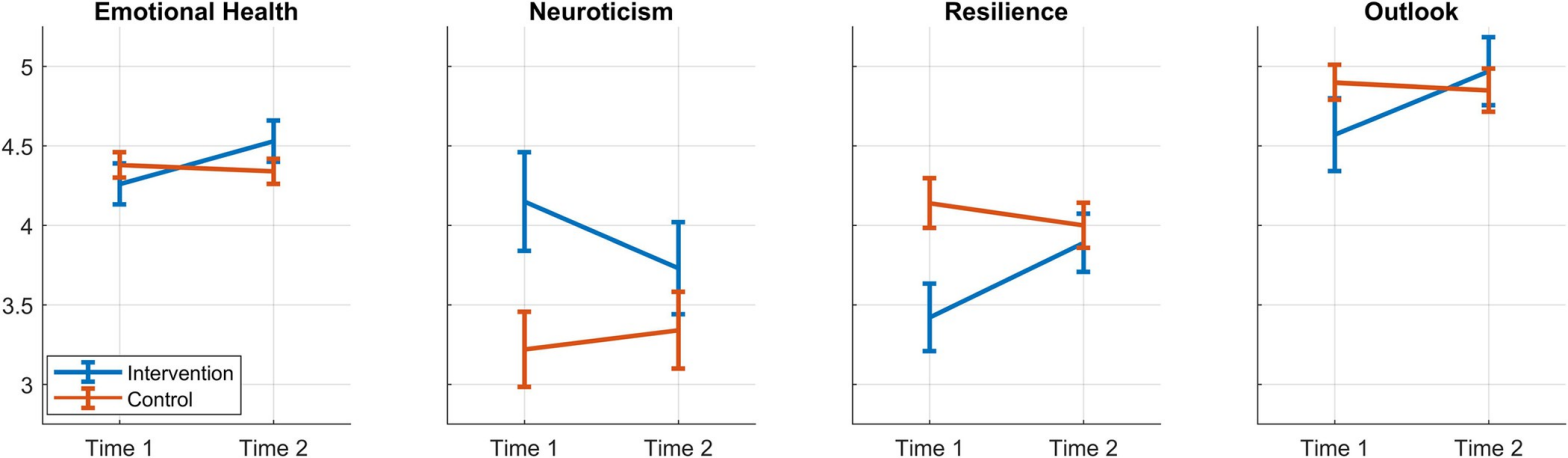
Phase 1 significant effects. Graphical representations of significant effects observed between pre- and post-test means in four measures for intervention and control groups. The vertical bars represent standard error of the mean.

To analyze the data, Repeated Measures ANOVA’s (RMANOVA’s) were conducted for each outcome variable, with Time (Time 1 and Time 2) specified as the within-subject factor and Group (intervention vs. control) specified as the between-group factor. Pre- and post-test scores for each group are presented in Table 1, as are the results of the RMANOVA’s. Fig 2 shows comparisons of the intervention and control groups for those results which were statistically significant (*p*-values reported above).

**Table 1:** Phase 1 pre- and post-test Means (*M*), Standard Deviations (*SD*), between group effect sizes (*d*), and RMANOVA results (*F*-value, *p*-value) for intervention and control groups.

	Intervention		Wait-list Control				
Variable	Pre-test *M* (*SD*)	Post-test *M* (*SD*)	Pre-test *M* (*SD*)	Post-test *M* (*SD*)	Between group *d*	Time x Group *F*-value	Time x Group *p*-value
ESQ—Overall	4.26 (0.63)	4.53 (0.63)	4.38 (0.47)	4.34 (0.46)	0.52	10.73	0.002**
ESQ—Outlook	4.57 (1.12)	4.97 (1.05)	4.90 (0.64)	4.85 (0.79)	0.45	8.92	0.004**
ESQ—Resilience	3.42 (1.04)	3.89 (0.90)	4.14 (0.91)	4.00 (0.83)	0.65	11.56	0.001**
ESQ—Social Intuition	4.89 (0.71)	4.96 (0.89)	4.51 (0.85)	4.50 (0.96)	0.10	0.35	0.554
ESQ—Self-Awareness	4.64 (0.94)	4.91 (0.94)	4.76 (0.66)	4.70 (0.62)	0.38	3.68	0.060
ESQ—Sensitivity to Context	4.66 (1.01)	4.85 (1.02)	4.46 (1.03)	4.47 (0.92)	0.18	1.61	0.210
ESQ—Attention	3.41 (1.11)	3.60 (1.07)	3.52 (1.03)	3.50 (1.10)	0.19	1.53	0.222
TIPI—Extraversion	3.56 (1.42)	3.83 (1.52)	3.91 (1.43)	3.90 (1.56)	0.19	1.93	0.170
TIPI—Neuroticism	4.15 (1.52)	3.73 (1.42)	3.22 (1.38)	3.34 (1.41)	-0.37	4.70	0.034*
TIPI—Agreeableness	4.88 (0.89)	5.00 (1.22)	4.50 (1.22)	4.76 (1.16)	-0.11	0.50	0.482
TIPI—Conscientiousness	5.21 (1.04)	5.02 (1.14)	4.84 (1.34)	4.84 (1.50)	-0.17	0.66	0.420
TIPI—Openness to Experience	5.21 (1.16)	5.19 (1.11)	5.37 (0.96)	5.24 (1.00)	0.12	0.27	0.603
PANAS—Positive Affect	3.21 (0.94)	3.48 (0.80)	3.36 (0.68)	3.29 (0.71)	0.41	3.57	0.064
PANAS—Negative Affect	2.23 (0.82)	2.24 (0.96)	2.04 (0.71)	2.06 (0.67)	-0.03	0.01	0.941
Physical Symptoms (CHIPS)	5.88 (4.33)	4.58 (3.39)	4.56 (3.35)	4.77 (2.72)	-0.40	2.12	0.151
Mindfulness (MAAS)	5.04 (0.71)	5.29 (0.68)	4.83 (0.66)	4.79 (0.69)	0.42	1.95	0.168
FFMQ-SF—Overall	4.15 (0.79)	4.36 (0.76)	4.19 (0.57)	4.18 (0.54)	0.29	1.89	0.175
FFMQ-SF—Observe	4.70 (1.11)	4.96 (0.99)	4.45 (1.11)	4.43 (1.10)	0.26	0.86	0.359
FFMQ-SF—Describe	4.42 (1.22)	4.68 (1.13)	4.46 (0.96)	4.64 (0.93)	0.04	0.19	0.666
FFMQ-SF—Act with Awareness	4.67 (1.01)	4.68 (1.15)	4.31 (1.21)	4.16 (1.17)	0.13	0.32	0.572
FFMQ-SF—Non-judge	3.46 (0.94)	3.68 (1.34)	3.73 (1.19)	3.52 (1.13)	0.37	2.18	0.146
FFMQ-SF—Non-react	3.60 (1.28)	3.93 (1.25)	4.03 (1.06)	4.20 (1.02)	0.10	0.35	0.558
Research Satisfaction	4.23 (1.11)	4.53 (1.15)	4.56 (1.05)	4.54 (1.32)	0.28	2.08	0.155
Contributive Desire	5.88 (0.79)	5.75 (0.96)	5.75 (0.95)	5.56 (1.00)	0.04	0.14	0.711

ESQ = Emotional Style Questionnaire. TIPI = Ten Item Personality Inventory. PANAS = Positive and Negative Affect Schedule. CHIPS = Cohen-Hoberman Inventory of Physical Symptoms. MAAS = Mindful Attention and Awareness Scale. FFMQ-SF = Five Facet Mindfulness Questionnaire-Short Form. For each of the measures, the sample size varied between 23–24 for the intervention group and 33–34 for the control group.

* P < 0.05.

** P < 0.01.

We also calculated Cohen’s *d* for the pre- to post- changes for both the intervention and the control group. Cohen’s *d* is a standardized measure of effect size, identifying the difference between two means in standard deviation units. To help interpret the magnitude of the study effects, Table 1 includes a column titled “between group *d*”. Between group *d* was computed by subtracting the pre- to post-test Cohen’s *d* for the control group from the pre- to post-test Cohen’s *d* for the intervention group [48]. Thus, larger absolute values of this statistic indicate larger changes favoring the intervention group relative to the control group.

The intercorrelations between all the measures used in the study, at pre-test and post-test separately, are provided in S2 and S3 Tables. Details on these results can be found in S1 Results.

Even though only a small number of our outcome variables yielded significant Group by Time interactions, overall we had a very consistent pattern of change favoring the intervention group.

### Phase 1: Qualitative measures

The qualitative summative survey was conducted approximately five months after the intervention group and one month after the control group had completed the training. The assessment was completed by 35 participants. It posed several open-ended questions, including “In what ways has the training impacted your research and other professional work?” Although not all respondents indicated that they had noticed changes, many provided exceptionally compelling responses indicating how the mindfulness practices were helping them better manage stress, regulate their emotions, ease anxiety, lessen anger, be more focused and efficient in their work, better handle interpersonal interactions, and deal with research setbacks in a positive manner (see S4 Table). Participants also commented on how the mindfulness training had improved their ability to work more effectively with other members of their research group. Sample comments include, “It exposed me to a new way of approaching situations.”; “More willing to consider and test new ideas instead of dismissing them.”; and “Making me a better listener and more active participant in communication with fewer distractions.” At the time of the summative survey, 65% of respondents reported that they were currently practicing mindfulness activities at least weekly (see Fig 4 for further detail).

### Phase 2: Quantitative measures

In Year 1 of Phase 2, a combined total of 90 participants completed both the pre- and post-test surveys and 89 passed built-in attention checks. The summative survey, conducted after the training had concluded for both the intervention and control groups, was completed by a combined total of 60 participants, and 44 went on to complete the final summative survey after Year 2. In Year 2, a combined total of 69 participants completed both the pre- and post-test surveys and 68 passed built-in attention checks. The summative survey was completed by a combined total of 43 participants.

The majority of the participants in Phase 2 (129 out of 156 respondents) had little to some familiarity with mindfulness prior to the study. 14 reported no familiarity at all and 13 reported “a great deal” of familiarity.

The data analytic procedures for Phase 2 were identical to those of Phase 1. We conducted Repeated Measures ANOVA’s (RMANOVA’s) for each outcome variable, with Time (Time 1 and Time 2) specified as the within-subject factor and Group (intervention vs. control) specified as the between-group factor. Given our relatively low sample sizes per university, we combined the samples from both universities and analyzed them together. This decision was justified by the absence of any systematic differences between the results of the two samples, as evinced by a lack of significant three-way interactions (i.e., Group x Time x University) when University was included as a between-group factor in our RMANOVA’s.

**Fig 3:**
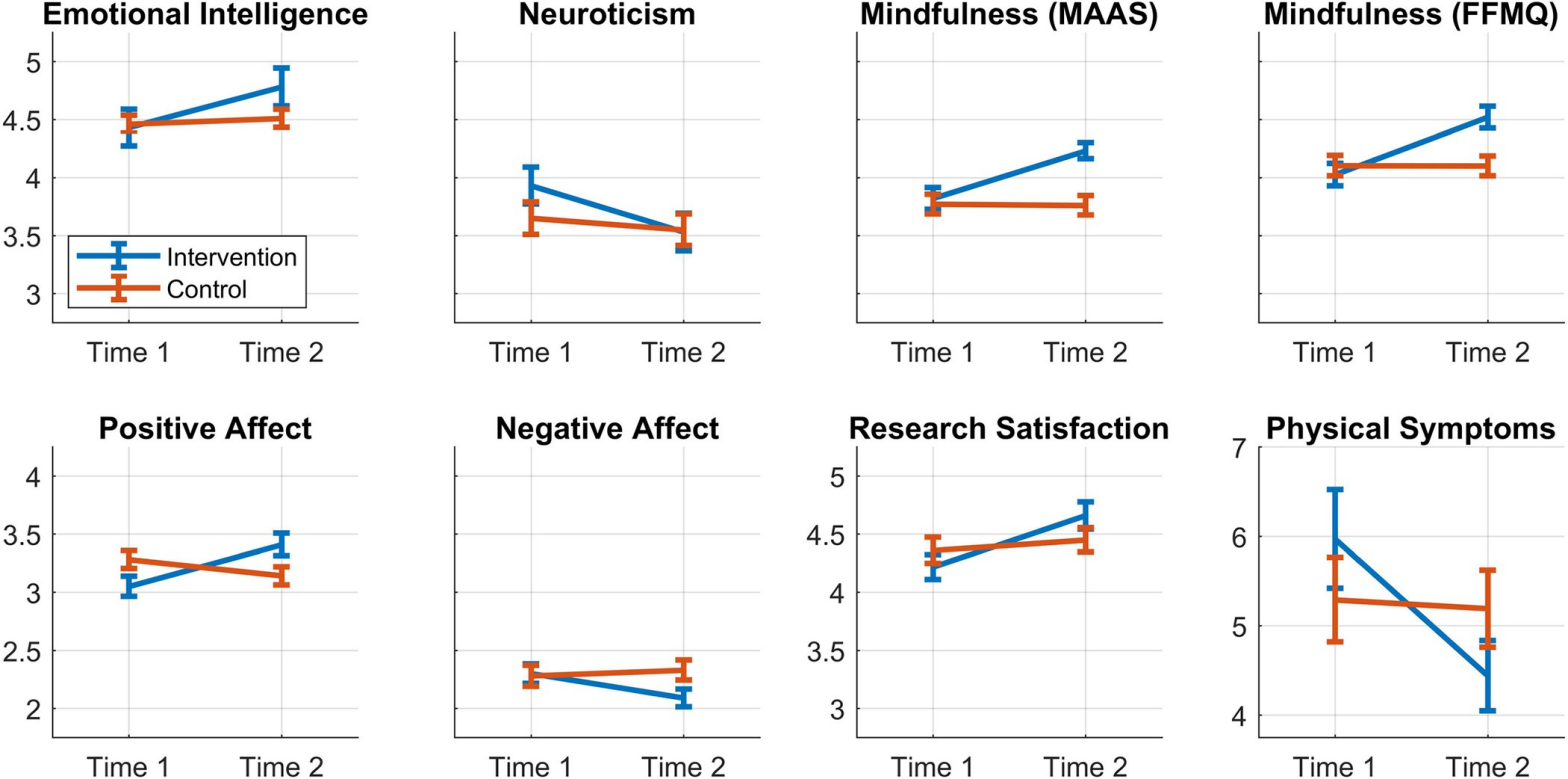
Phase 2 significant effects. Graphical representations of eight significant effects observed between pre- and post-test means for intervention and control groups. The vertical bars represent standard error of the mean. Phase 2 Year 1 and Year 2 are combined.

Pre- and post-test scores for Phase 2 are presented in Table 2 (Year 1 and Year 2 combined), as are the results of the RMANOVA’s. Details of the individual years of the study are provided in S7 and S8 Tables. Fig 3 shows comparisons of the intervention and control groups for those results which were statistically significant (*p*-values reported above). As above, we calculated Cohen’s *d* for the pre- to post- changes for both the intervention and the control group. The intercorrelations between all the measures used in the study, at pre-test and post-test separately, are provided in S9 and S10 Tables.

**Table 2:** Phase 2 pre- and post-test Means (*M*), Standard Deviations (*SD*), between group effect sizes (*d*), and RMANOVA results (*F*-value, *p*-value) for intervention and control groups (Year 1 and Year 2 combined).

	Intervention		Wait-list Control				
Variable	Pre-test *M* (*SD*)	Post-test *M* (*SD*)	Pre-test *M* (*SD*)	Post-test *M* (*SD*)	Between group *d*	Time x Group *F*-value	Time x Group *p*-value
ESQ—Overall	4.43 (0.75)	4.78 (0.72)	4.46 (0.71)	4.51 (0.72)	0.41	14.37	< 0.001**
ESQ—Outlook	4.66 (1.22)	5.05 (1.18)	4.63 (1.20)	4.71 (1.10)	0.26	4.75	0.031*
ESQ—Resilience	3.93 (1.16)	4.40 (1.13)	4.07 (1.21)	4.25 (1.16)	0.26	3.74	0.055
ESQ—Social Intuition	4.95 (1.13)	5.11 (1.09)	4.74 (1.10)	4.82 (1.11)	0.07	0.41	0.524
ESQ—Self-Awareness	4.45 (1.22)	4.92 (0.99)	4.65 (1.28)	4.58 (1.19)	0.47	12.79	< 0.001**
ESQ—Sensitivity to Context	4.95 (1.22)	5.21 (1.06)	4.76 (1.00)	4.93 (1.04)	0.06	0.40	0.527
ESQ—Attention	3.62 (1.29)	4.00 (1.15)	3.92 (1.22)	3.79 (1.16)	0.42	14.39	< 0.001**
TIPI—Extraversion	3.82 (1.51)	3.90 (1.38)	3.84 (1.48)	4.04 (1.36)	-0.08	0.59	0.444
TIPI—Neuroticism	3.93 (1.35)	3.53 (1.39)	3.65 (1.29)	3.55 (1.25)	-0.21	4.88	0.029*
TIPI—Agreeableness	5.02 (1.16)	5.07 (1.21)	4.63 (1.07)	4.76 (1.07)	-0.08	0.39	0.532
TIPI—Conscientiousness	5.21 (1.11)	5.21 (1.26)	4.99 (1.36)	4.91 (1.26)	0.06	0.42	0.516
TIPI—Openness to Experience	5.21 (0.96)	5.34 (1.12)	5.02 (1.11)	5.17 (0.98)	-0.02	0.03	0.874
PANAS—Positive Affect	3.05 (0.74)	3.41 (0.84)	3.28 (0.71)	3.14 (0.71)	0.66	25.41	< 0.001**
PANAS—Negative Affect	2.30 (0.71)	2.09 (0.66)	2.28 (0.84)	2.33 (0.79)	-0.36	6.85	0.010*
Physical Symptoms (CHIPS)	5.97 (4.71)	4.44 (3.35)	5.29 (4.32)	5.19 (3.96)	-0.35	5.06	0.026*
Mindfulness (MAAS)	3.82 (0.80)	4.23 (0.58)	3.77 (0.78)	3.76 (0.77)	0.59	16.11	< 0.001**
FFMQ-SF—Overall	4.02 (0.82)	4.52 (0.80)	4.10 (0.81)	4.10 (0.77)	0.62	25.55	< 0.001**
FFMQ-SF—Observe	4.94 (1.20)	5.29 (1.12)	4.65 (1.12)	4.50 (1.12)	0.44	11.33	0.001**
FFMQ-SF—Describe	4.05 (1.28)	4.65 (1.09)	4.09 (1.40)	4.20 (1.34)	0.43	8.75	0.004**
FFMQ-SF—Act with Awareness	4.42 (1.40)	4.88 (1.06)	4.39 (1.33)	4.26 (1.31)	0.47	11.53	0.001**
FFMQ-SF—Non-judge	3.36 (1.18)	3.80 (1.33)	3.64 (1.15)	3.72 (1.16)	0.28	4.52	0.035*
FFMQ-SF—Non-react	3.54 (1.21)	4.16 (1.17)	3.85 (1.18)	3.91 (1.12)	0.47	12.80	< 0.001**
Research Satisfaction	4.22 (0.91)	4.66 (0.99)	4.36 (1.05)	4.45 (0.96)	0.37	6.84	0.010*
Contributive Desire	5.66 (1.17)	5.70 (1.16)	5.63 (0.94)	5.65 (0.99)	0.00	0.00	0.974

ESQ = Emotional Style Questionnaire. TIPI = Ten Item Personality Inventory. PANAS = Positive and Negative Affect Schedule. CHIPS = Cohen-Hoberman Inventory of Physical Symptoms. MAAS = Mindful Attention and Awareness Scale. FFMQ-SF = Five Facet Mindfulness Questionnaire-Short Form For each of the measures, the sample size varied between 72–73 for the intervention groups and 83–84 for the control groups.

* *p* < 0.05.

** *p* < 0.01.

We break down the presentation of Phase 2 results by Year 1 and Year 2. As the effects we observed were not qualified by year (i.e., no significant Group x Time x Year interactions), we also report the results of the analyses in the combined sample (N = 157) to provide a more comprehensive picture for the reader.

Before running the analyses, independent t-tests were conducted to compare groups at baseline (i.e., at pre-test) for demographic variables and outcome measures. In Year 1, a significant difference was observed for the Neuroticism variable, such that those in the intervention group scored significantly higher in Neuroticism at the pre-test than those in the waitlist control group (*p =* 0.028). In Year 2, a significant baseline difference was noted for Agreeableness, with the intervention group scoring higher in Agreeableness than the control group (*p =* 0.021). When both years were combined, this baseline difference in Agreeableness persisted (*p* = 0.031). In this combined sample, a baseline difference for Positive Affect was also observed, with those in the intervention group reporting initially lower levels of Positive Affect than those in the control group (*p* = 0.042).

There were no significant differences for any of the other variables for Phase 2 (*p*’s > 0.05) when analyzed in aggregate. Further analyses revealed that for a few of the variables, the 3-way Time x Group x Gender interaction was significant, suggesting that the significant Time x Group interactions we observed were qualified by Gender. In those cases, both men and women benefited from the intervention, although women benefited slightly more.

Overall, we had a very consistent pattern of change favoring the intervention group, particularly when it comes to variables related to emotional well-being and mindfulness. The effects were more apparent in the combined, larger sample.

Details on these results can be found in the S4 Results.

### Phase 2: Qualitative measures

The qualitative summative survey was conducted approximately 5 months after the intervention group and 1 month after the control group had completed the training in Year 1 of Phase 2. The Summative Survey for Year 2 cohorts was impacted by the pandemic in two ways: the control group received a hybrid training experience with the first five weeks in person, a pause of two weeks, then the remaining three weeks delivered online. Both the intervention and control groups completed the Summative Survey in the midst of the COVID-19 pandemic and the administration of this was conducted approximately twelve months after the intervention group and six months after the control group had completed the training.

The assessment was completed by 60 participants in Year 1 and 43 participants in Year 2. It included the same open-ended questions as those asked in Phase 1. The majority of participants noted positive changes echoing those reported in Phase 1 (see S11 and S12 Tables). Open-ended responses were consistent between the three Summative Surveys conducted. Several participants in Year 2 noted positive impacts of the training in relation to the pandemic, e.g., “…Overall, I think the training helped me to be more productive and avoid burnouts during these difficult pandemic times."

**Fig 4:**
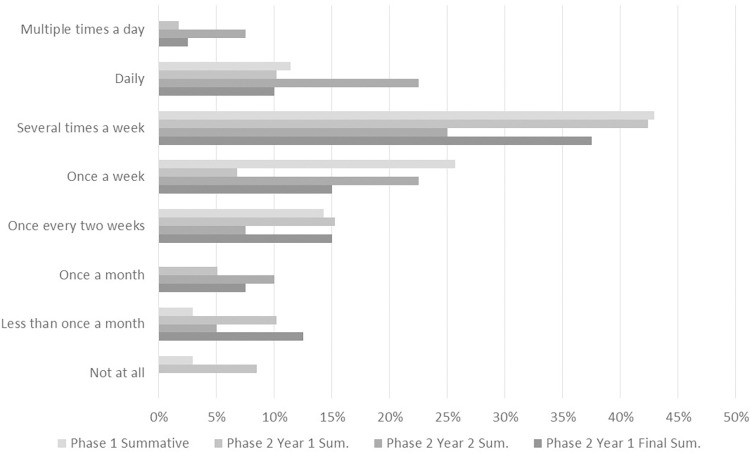
Ongoing mindfulness practice. Responses to the Summative Survey question “To what extent do you practice mindfulness activities (e.g., meditation, yoga, journaling, reflection, etc.) currently?”.

Combining Year 1 and Year 2 summative data, 68% of respondents reported that they were currently practicing mindfulness activities weekly or more frequently (see Fig 4 for further detail). Reported practice frequency covered a wide range: 13 at once a week, 35 at several times a week, 15 at daily, and 4 at multiple times a day.

The Final Summative Survey, completed by 44 of the Phase 2 Year 1 participants was intended to be a more longitudinal measure of the training. This was conducted in November of 2020, approximately 24 months after the intervention group and 18 months after the control group had completed the training in Year 1. Several responses directly mentioned the “pandemic” and “COVID”, with some indicating that the impacts of the pandemic made it difficult for them to separate out the impacts of the training, while others indicated more direct positive influence of the training relative to the pandemic (e.g. “The training has helped especially during COVID era back to back zoom calls. While I can relax and work from home, the back to back zoom calls definitely takes a toll so the STOP technique and other breathing techniques have helped me relax in between meetings….”). See S13 Table for further details.

Open-ended responses were relatively consistent between the three shorter term Summative Surveys and the more longitudinal Final Summative Survey. Responses to the questions “In what ways has the training impacted your research and other professional work?” and “In what ways has the training impacted your personal life?” were less positive for the Year 1 Final Summative Survey than the responses for the Summative Survey of the Year 2 cohort in Phase 2. These two groups provide the most relevant comparisons because the assessments were both conducted in November of 2020 (during the pandemic) and comparisons can be made between responses from participants who had most recently completed the training (Phase 2 Year 2 Summative) to those from participants who had completed the training one year prior (Phase 2 Year 1 Final Summative).

Participants’ prior exposure to mindfulness practice covered a wide range. Some participants reported prior use of practices such as meditation, yoga, and/or prayer. However, none had received prior training on the breadth of topics covered nor the range of guided meditations provided by the training in this study. The training included mindfulness practices spanning a range of tools for participants to use in their own independent mindfulness practice.

Over half of the Phase 1 participants reported at the point of their Summative Survey that they continue to practice mindfulness activities daily or several times a week with an additional quarter practicing once a week (Fig 4). The amount of time they reported devoting to mindfulness activities weekly varied, but the average of all responses was approximately 1 hour/week and the median was 35 minutes.

Fig 4 further summarizes the ongoing practice of Phase 2 participants at both the Summative Survey and Final Summative Survey time points. 20 of the participants reported that they continue to practice mindfulness activities daily or several times a week with an additional 6 participants practicing once a week 18–24 months after the training was completed.

## Discussion

The purpose of this research was to explore the impact of a mindfulness training on engineering graduate students, with a particular focus on how this training affected their well-being and capacity for research. During the course of the two studies comprising three years, where each group engaged in training for eight weeks in the middle of a semester, the intervention groups reported significantly improved emotional well-being at the end of the training. In contrast, the control groups consistently held steady or dropped on their well-being during that same time frame. While statistical significance was not observed across each cohort (e.g., intervention/control pair) for all measures, the combined data across both studies, two institutions, and multiple years of Phase 2 produced consistent and robust findings. Together these findings provide compelling evidence of the efficacy of this type of training with engineering graduate students.

More specifically, both studies showed higher Emotional Health over time as measured by the ESQ, with significant effects observed for the Resilience and Outlook subscales in Phase 1 and the Attention, Self-Awareness, and Outlook subscales in Phase 2. Additionally, across both studies, participants in the intervention group perceived themselves as less neurotic over time. Following the trend seen in Phase 1, there were significantly higher levels of Positive Affect and significantly lower levels of Negative Affect as well as significant gains in mindful attention and awareness in the Phase 2 intervention groups. A significant increase in mindfulness was observed in the intervention groups of Phase 2 with all the subscales of the FFMQ-SF. This too is consistent with trends observed in Phase 1. Although the Research Satisfaction Scale did not reach significance in Phase 1, the intervention groups in Phase 2 reported statistically significant improvement in their satisfaction with their research across time. More extensive study of the impact on research satisfaction is warranted, especially given its links to persistence and achievement [9].

The improvements in emotional health and wellbeing observed are consistent with those reported in prior studies of similar interventions with postbaccalaureate students. Guided mindfulness practices in 8-week interventions in a similar age group of medical school students and psychology graduate students have been shown to reduce stress and anxiety [32, 35]. Additionally, prior research has shown evidence that attention is cultivated and enhanced with mindfulness training. Medical and nursing student participants who received 8-weeks of mindfulness-based stress reduction training, a similar age group of postbaccalaureate students to the study reported here, showed improvements in concentrative attention [22]. Meditation training was also shown to improve attention for undergraduate Chinese students after five days of training in comparison to relaxation training [28 Tang]. Consistent with the above quantitative findings, the open-ended responses collected in the summative surveys spoke to a better ability to regulate emotion and anger, decreased anxiety, and reduced stress. Of particular importance to the ability to conduct engineering research, the graduate student respondents cited increases in focus and efficiency, improved relations with research colleagues, and enhanced ability to handle research setbacks constructively. It is also encouraging that a substantial number of participants have reported that they continue to practice mindfulness 18–24 months after the program ended, although the average time spent per week did diminish.

Another notable finding of our study was that engineering graduate students were open to this type of training and ultimately expressed high levels of contentment with it. An inspection of qualitative responses to the summative survey revealed that participants appreciated the opportunity to devote time weekly to the in-class training where they would practice the meditation skills being learned. These weekly training sessions also provided them with an opportunity to connect with other graduate students. The small groups formed within each training group added an opportunity for regular check-ins with peers, which provided additional support and accountability. This social support aspect of the training could have presumably contributed to the positive outcomes observed in the intervention group. Overall, we consider the training’s positive reception among engineering graduate students a valuable learning from our study, given that this population might not necessarily be thought of as naturally drawn to this type of contemplative practice.

Although we did not select participants, we found post facto that women were substantially overrepresented (40% and 46% for Phase 1 and Phase 2) and domestic minority students slightly overrepresented (10% for both studies) among the participants in this study compared to graduate student enrollment in engineering departments at University A (22–25% female and 6–8% domestic minority, over the time frames of the two studies). International students were modestly underrepresented (36% and 39% for Phase 1 and Phase 2, respectively, compared to 41–42% over the time frames of the two studies). Although the overrepresentation of female and minority students allows for more robust conclusions over the breadth of engineering graduate student backgrounds, it does indicate that voluntary participation in such activities may be more challenging to achieve among other student populations. This may have ties to the differences seen in treatment seeking for mental health and related services seen across disciplines in undergraduate students [11].

Further analyses conducted with Phase 2 data revealed that for a few of the variables, the three-way Time x Group x Gender interaction was significant, suggesting that the significant Time x Group interactions we observed were qualified by Gender. Looking at those more closely, it appeared that in those cases although both men and women benefited from the intervention, women benefited slightly more. Whereas a recent meta-analysis (Bamber and Morpeth 2019) did not find evidence that gender moderated the effects regarding anxiety in the studies of mindfulness meditation on college student anxiety, there is other evidence in the literature that women may be more likely to benefit from mindfulness interventions [49, 50]. Gender differences in response to mindfulness-based training is an open area deserving further study.

We note some limitations of our study. One limitation is that despite the use of random assignment, we observed some baseline differences between the intervention and the control groups, although not consistently recurring between studies and years. These pre-existing differences invite caution when interpreting the results related to these measures in an individual study or year. Regression to the mean is a possibility that we cannot rule out with our study design. We also acknowledge the possibility of demand characteristic effects in the intervention group, especially with measures of mindfulness and the Emotional Style Questionnaire, which closely align with the training content. Yet another limitation is methodological: while we had a wait-list control group that allowed us to control for the passage of time, we did not have an active control group that could control for other non-specific training effects [51, 52]. Finally, we should also note that we ran a large number of statistical tests (given our large number of variables), which might have inflated the Type I error rate in our study. Notwithstanding these limitations and the need for further research, we believe that the present results justify the introduction of mindfulness-based programs as an effective and well-received method to improve graduate student well-being in engineering education. There were no statistically significant differences found between institutions, further supporting the robustness of the findings.

Mounting evidence points to the increasing prevalence of problematic mental health and well-being issues in the graduate student populations of our higher education institutions [2–4, 9]. Addressing these issues will require a variety of resources, as well as policy and practice changes. This study points to one type of practical intervention that can be implemented at the institution, college, department or program level to support well-being in the context of graduate education.

## Conclusion

The eight-week mindfulness-based training program provided to engineering graduate student participants was well-received and produced measurable positive impacts on emotional well-being. The improvements in emotional health were highly meaningful for a graduate student sample, where high levels of stress and anxiety are commonly reported. Furthermore, the positive implications for mindfulness and research satisfaction are highly encouraging. These findings motivate broader utilization of mindfulness-based contemplative training to enhance both the personal and professional lives of engineering graduate students.

## Supporting information

S1 Methods Supplementary methods. (DOCX)

S1 Results Phase 1 supplementary results. (DOCX)

S2 Results Additional supplementary results. (DOCX)

S3 Results Additional summative survey findings. (DOCX)

S4 Results Phase 2 supplementary results. (DOCX)

S1 References Supplementary references. (DOCX)

S1 Table Outline of the training curriculum developed by Healthy Minds Innovations. Page numbers refer to the relevant sections of The Emotional Life of Your Brain for each week of training (Davidson and Begley 2012).
(DOCX)

S2 Table Intercorrelations between measures at pre-test for Phase 1. (DOCX)

S3 Table Intercorrelations between measures at post-test for Phase 1. (DOCX)

S4 Table Summative survey results and representative responses for Phase 1 (n = 35). (DOCX)

S5 Table Summary of participant and completion statistics for Phase 2—Year 1. (DOCX)

S6 Table Summary of participant and completion statistics for Phase 2—Year 2. (DOCX)

S7 Table Phase 2 Year 1 pre- and post-test Means (M), Standard Deviations (SD), between group effect sizes (d), and RMANOVA results (F-value, p-value) for intervention and control groups. ESQ = Emotional Style Questionnaire. TIPI = Ten Item Personality Inventory. PANAS = Positive and Negative Affect Schedule. CHIPS = Cohen-Hoberman Inventory of Physical Symptoms. MAAS = Mindful Attention and Awareness Scale. FFMQ-SF = Five Facet Mindfulness Questionnaire-Short Form. AUT = Alternate Uses Task. For each of the measures, the sample size varied between 37–38 for the intervention group and 50–51 for the control group. * p < 0.05. ** p < 0.01.
(DOCX)

S8 Table Phase 2 Year 2 pre- and post-test Means (M), Standard Deviations (SD), between group effect sizes (d), and RMANOVA results (F-value, p-value) for intervention and control groups. ESQ = Emotional Style Questionnaire. TIPI = Ten Item Personality Inventory. PANAS = Positive and Negative Affect Schedule. CHIPS = Cohen-Hoberman Inventory of Physical Symptoms. MAAS = Mindful Attention and Awareness Scale. FFMQ-SF = Five Facet Mindfulness Questionnaire-Short Form. AUT = Alternate Uses Task. For each of the measures, the sample size was 35 for the intervention group and 33 for the control group. * p < 0.05. ** p < 0.01.
(DOCX)

S9 Table Intercorrelations between measures at pre-test for Phase 2. (DOCX)

S10 Table Intercorrelations between measures at post-test for Phase 2. (DOCX)

S11 Table Summative survey results and representative responses for Phase 2 Year 1 (n = 49). (DOCX)

S12 Table Summative survey results and representative responses for Phase 2 Year 2 (n = 43). (DOCX)

S13 Table Summative survey results and representative responses for phase 2 Year 1 final summative (n = 44). (DOCX)

S1 Fig Impact on research. Summative Data Comparison Across Years for Positive, Negative and Neutral Responses to “In what ways has the training impacted your research and other professional work?”.
(DOCX)

S2 Fig Impact on personal life. Summative Data Comparison Across Years for Positive, Negative and Neutral Responses to “In what ways has the training impacted your personal life?”.
(DOCX)

S3 Fig Recommend training to others. Summative Data Comparison across Years for Yes, Maybe, and No Responses to “Would you recommend this training to other engineering graduate students?”.
(DOCX)
