# Supplementary material for: Cultivating well-being in engineering graduate students through mindfulness training
Source: PLoS One. 2023 Mar 22;18(3):e0281994. doi: 10.1371/journal.pone.0281994 (PMC10032494; doi:10.1371/journal.pone.0281994)
Supplement: S1 Methods — (DOCX) [file pone.0281994.s001.docx]

**S2 Supplementary Methods**

**Satisfaction with Research Scale**

Please indicate your agreement with the following statements using the scale below. Try to be as honest and spontaneous in your responses as you can.

1 = Strongly disagree

2 = Mostly disagree

3 = Somewhat disagree

4 = Neither agree nor disagree

5 = Somewhat agree

6 = Mostly agree

7 = Strongly agree

1. I am satisfied with how my research is progressing these days.
2. I feel efficient and productive in my research activities these days.
3. I feel frustrated with my research these days. (r)
4. I feel like I’m developing my research skills.
5. I feel creative and innovative in my research these days.
6. I don’t feel like I’m able to come up with new research ideas these days. (r)
7. I feel like I’m able to overcome obstacles in my research these days.
8. I feel excited about my research these days.

**Contributive Desire Scale**

Please indicate your agreement with the following statements using the scale below. Try to be as honest and spontaneous in your responses as you can.

1 = Strongly disagree

2 = Mostly disagree

3 = Somewhat disagree

4 = Neither agree nor disagree

5 = Somewhat agree

6 = Mostly agree

7 = Strongly agree

1. I am motivated to use my knowledge and skills to make a difference in people’s lives.
2. I am driven to do things that have a positive impact on others.
3. Contributing to the betterment of society is not necessarily an important value to me. (r)
4. I feel a responsibility to do things to improve the well-being of people.
5. It is not that important to me to see my work have a concrete positive impact on people’s lives. (r)

**Creativity Characteristics Scale**

Please indicate your agreement with the following statements using the scale below. Try to be as honest and spontaneous in your responses as you can.

1 = Strongly disagree

2 = Mostly disagree

3 = Somewhat disagree

4 = Neither agree nor disagree

5 = Somewhat agree

6 = Mostly agree

7 = Strongly agree

1. I am good at coming up with new and original ideas.
2. I am curious about a wide variety of topics.
3. I am not good at dealing with uncertainties or ambiguities. (r)
4. When I come up with a good idea, I am good at translating it into action.
5. I get uncomfortable when things do not go according to plan. (r)
6. I like to solve complex problems.
7. I am never bored.
8. I am not particularly interested in the arts. (r)
9. I love to think up new ways of doing things.
10. I find the world a very interesting place.
11. I try to please everyone. (r)
12. I dig deeper into ideas that I’m interested in.
13. I finish what I start, despite obstacles in the way.
14. I ask questions that nobody else does.
15. I do not like reading or hearing opinions that go against my way of thinking. (r)
