# Supplementary material for: Cultivating well-being in engineering graduate students through mindfulness training
Source: PLoS One. 2023 Mar 22;18(3):e0281994. doi: 10.1371/journal.pone.0281994 (PMC10032494; doi:10.1371/journal.pone.0281994)
Supplement: S1 Results — (DOCX) [file pone.0281994.s002.docx]

**S5 Phase 1 Supplementary Results**

***Emotional Style Questionnaire (ESQ)***

The RMANOVA yielded a significant Time by Group interaction for the overall questionnaire, denoting improved Emotional Health for the intervention group relative to the control group, *F*(1, 56) = 10.73, *p* = 0.002. Paired *t*-tests conducted post hoc revealed a significant increase in emotional health in the intervention group, *t*(23) = -2.66, *p* = 0.014, *d* = 0.42, but not in the control group, *t*(33) = 1.20, *P* = 0.240, *d* = -0.10. The between-groups effect (*d* = 0.52) was medium in size. Among the subscales of the ESQ, *Resilience*, *F*(1, 56) = 11.56, *p* = 0.001, *Outlook*, *F*(1, 56) = 8.92, *p* = 0.004, and *Self-Awareness*, *F*(1, 56) = 3.68, *p* = 0.060 exhibited the largest improvements. The between group *d*’s were 0.65 for *Resilience*, 0.45 for *Outlook*, and 0.38 for *Self-Awareness*.

***Ten Item Personality Inventory (TIPI)***

Among the different facets of the TIPI, we noted a significant Time by Group interaction for *Neuroticism*, *F*(1, 56) = 4.70, *p* = 0.034. As revealed by post-hoc t-tests, there was a non-significant trend for the participants in the intervention group to perceive themselves as less neurotic over time, *t*(23) = 1.78, *p* = 0.089, *d* = -0.28 whereas no such trend was observed in the control group, *t*(33) = -0.94, *p* = 0.353, *d* = 0.08. The between group difference was small-to-medium (*d* = -0.37).

***Positive and Negative Affect Schedule (PANAS)***

The Positive Affect subscale of the PANAS yielded a marginally significant Time by Group interaction, *F*(1, 56) = 3.57, *p* = 0.064, indicating that the training led to a trend toward increased positive affect among the intervention group (between group *d* = 0.41). No significant effect was noted for the Negative Affect subscale (*p* = 0.941).

***Cohen-Hoberman Inventory of Physical Symptoms (CHIPS)***

Although the Time by Group interaction for CHIPS did not reach significance, *F*(1, 56) = 2.12, *p* = 0.151, inspection of effect sizes supported a small-to-medium size reduction in health symptoms favoring the intervention group (between group *d* = -0.40).

***Mindful Attention and Awareness Scale (MAAS)***

The RMANOVA results for the MAAS did not reach significance, *F*(1, 56) = 1.95, *p* = 0.168. However, an inspection of effect sizes again revealed a small-to-medium size increase in mindful attention and awareness favoring the intervention group (between group *d* = 0.42).

***Five Facet Mindfulness Questionnaire - Short Form (FFMQ-SF)***

The Time by Group interaction for the overall FFMQ-SF score did not reach significance, *F*(1, 55) = 1.89, *p* = 0.175. Nonetheless, as with the MAAS, there was an effect favoring the intervention group (between group *d* = 0.29). Further analyses revealed that the largest improvements were observed for the *Non-Judge* (between group *d* = 0.37) subscale and the *Observe* subscale (between group *d* = 0.26).

***Research Satisfaction Scale***

The RMANOVA results for the Research Satisfaction Scale did not reach significance, *F*(1, 55) = 2.08, *p* = 0.155. Yet there was a small effect (between group *d* = 0.28) favoring the intervention group.

***Contributive Desire Scale***

No significant Time by Group interaction was noted for the Contributive Desire Scale (*p* = 0.711).

Results for four additional scales are reported in the Additional Supplementary Results for Phase 1: Flourishing Scale, Perceived Stress Scale, Alternate Uses Task, and the Creativity Characteristics Scale.
