# Supplementary material for: Cultivating well-being in engineering graduate students through mindfulness training
Source: PLoS One. 2023 Mar 22;18(3):e0281994. doi: 10.1371/journal.pone.0281994 (PMC10032494; doi:10.1371/journal.pone.0281994)
Supplement: S2 Results — (DOCX) [file pone.0281994.s003.docx]

**S6 Additional Supplementary Results**

**Flourishing Scale [Phase 1]**

The Flourishing Scale is an eight-item measure capturing participants’ self-perceived success in domains important to well-being, such as relationships, self-esteem, and optimism (Diener et al. 2010). Participants indicated on a Likert scale ranging from 1 (*strongly disagree*) to 7 (*strongly agree*) how much they agree with statements such as “I lead a purposeful and meaningful life” or “My social relationships are supportive and rewarding.” (Cronbach’s alpha = .83 at pre-test and .91 at post-test).

No significant Time by Group interaction was observed for the Flourishing Scale (*p* = 0.303).

**Perceived Stress Scale [Phase 1]**

We used the 10-item Perceived Stress Scale, which assesses the degree to which a person appraises their life as stressful (i.e., difficult to predict, control, and cope with) (Cohen and Williamson 1988). On a scale ranging from 1 (*never*) to 5 (*very often*) participants responded to questions such as “In the last two weeks, how often have you felt that you were unable to control the important things in your life?” or “In the last two weeks, how often have you felt that you were on top of things?”. (Cronbach’s alpha = .89 at pre-test and .87 at post-test).

The Perceived Stress Scale yielded no significant Time by Group interaction (*p* = 0.816).

**Creativity Characteristics Scale [Phase 1 and Phase 2]**

To assess how much participants possessed personality characteristics associated with creativity and innovation, we developed 15 items (please see Supplementary Methods above). These items were generated after surveying relevant literature and aimed to capture self-perceptions of originality (e.g., “I am good at coming up with new and original ideas”), curiosity (e.g., “I am curious about a wide variety of topics”), comfort with ambiguities (e.g., “I am not good at dealing with uncertainties or ambiguities”), and ability to turn one’s ideas into action (e.g., “When I come up with a good idea, I am good at translating it into action”). Participants responded to these statements on a scale ranging from 1 (*strongly disagree*) to 7 (*strongly agree*). Internal reliability was acceptable (Cronbach’s alpha of .71 at pre-test and .75 at post-test in Phase 1).

In Phase 1, we observed a significant Time by Group interaction for the Creativity Characteristics Scale, *F*(1, 55) = 4.47, *p* = 0.039. According to paired *t*-tests conducted post-hoc, the intervention group showed a non-significant trend toward increased endorsement of creativity characteristics, *t*(23) = -1.72, *p* = 0.100, *d* = 0.21. However, no such effect was observed in the control group, *t*(32) = 1.16, *p* = 0.254, *d* = -0.11. The between-groups effect was small-to-medium in size (*d* = 0.32).

In Phase 2, we again observed a significant Time by Group interaction for the Creativity Characteristics Scale, *F*(1, 155) = 3.91, *p* = 0.050. According to paired *t*-tests conducted post-hoc, the intervention group increased in their endorsement of creativity characteristics, *t*(72) = -2.89, *p* = 0.005, *d* = 0.23. However, no such effect was observed in the control group, *t*(83) = -0.40, *p* = 0.693, *d* = 0.03. The between-groups effect size was *d* = 0.20.

**Alternate Uses Task (AUT) [Phase 1 and Phase 2]**

To capture participants’ creativity through behavioral means, we turned to the Alternate Uses Task. In this commonly employed measure of divergent creativity, participants are asked to think of as many alternate uses of a common object as possible within a fixed period of time (Guilford 1967).

Participants were first explained the task, given an example (“alternate uses for a ping pong ball”) and on the next page instructed to write down as many uses of a “paper clip” or a “brick” in two minutes as possible. After two minutes the page automatically advanced to the next page. For counterbalancing purposes across the pre-test and post-test, half of the participants had “paper clip” as their object during the pre-test, and the other half had “brick”. In the post-test, participants received the opposite object. Typical metrics for evaluating the responses of this divergent-thinking activity include quantity, quality, variety, and novelty (Smith and Ward 2012; Silvia 2015). To quantify responses on this task, we counted all responses a participant gave.

In Phase 1, the Time by Group interaction for the Alternate Uses Task did not reach significance, *F*(1, 55) = 1.63, *p* = 0.21.

In Phase 2, the Time by Group interaction for the Alternate Uses Task did not reach significance either, *F*(1, 155) = 0.28, *p* = 0.60.

**RAT (Remote Associates Test) [Phase 2]**

The Remote Associates Test has been developed as a measure of creative thought (Mednick 1962). The test items consist of three words (e.g., fox/man/peep) that are all associated with a common solution word (“hole”) in some way. In our study participants received 8 RAT problems at both pre-test and the post-test and were given a total of 5 minutes to solve them. These problems were taken from the normative set of Bowden and Jung-Beeman (Bowden and Jung-Beeman 2003). We created two sets of 8 RAT problems that were matched for difficulty. For counterbalancing purposes across the pre-test and post-test, half of the participants were given the first set at the pre-test, and half of the participants the second set. In the post-test, participants received the other set.

The Time by Group interaction for the RAT was not significant, *F*(1, 155) = 1.64, *p* = 0.20.

**Pattern Meanings Test [Phase 2]**

Pattern meanings test, originally developed by Wallach and Kogan (Wallach and Kogan 1965) measures creativity. It consists of abstract patterns (e.g., a triangle with small circles along each side), and participants are asked to interpret this pattern. In our study we used only one pattern, taken from Doherty and Mair (Doherty and Mair 2012) and asked participants to write down all of the things they think the pattern could be, or that it reminds them of. They had 2 minutes to respond to this question. As with the Alternate Uses Task and the Remote Associates Test, we had two versions of this pattern, which were each presented to only half of the participants and alternated at the pre-test and the post-test.

The Time by Group interaction for the Pattern Meanings Test was not significant, *F*(1, 153) = 1.14, *p* = 0.29.

**Depression, Anxiety, and Stress Scales (DASS-21) [Phase 2]**

To assess participants’ psychological distress, we used the short form version of the Depression Anxiety Stress Scales (DASS-21) (Henry and Crawford 2005). This is a 21-item scale that measures the dimensions of depression, anxiety, and stress, whereas the overall scale score is an indicator of overall psychological distress. Participants read statements such as “I felt that I had nothing to look forward to” or “I felt scared without any good reason”, and then indicated on a scale ranging from 1 (*did not apply to me at all*) to 4 (*applied to me very much or most of the time*) what their personal experience was over the last two weeks.

A significant Time by Group interaction was observed for the overall DASS-21 score benefiting the intervention group, *F*(1, 154) = 12.56, *p* = 0.001. Specifically, the intervention group experienced a significant decrease in their overall psychological distress, *t*(71) = 3.80, *p* < 0.001, *d* = -0.44, whereas the control group did not, *t*(83) = -0.72, *p* = 0.474, *d* = 0.05. Among the subscales of the DASS-21, *Depression*, *F*(1, 154) = 11.53, *p* = 0.001, and *Stress*, *F*(1, 154) = 10.73, *p* = 0.001 exhibited the largest effects. The Time by Group interaction for the *Anxiety* subscale did not reach significance (*p* = .151). The between group *d*’s were -0.51 for *Depression*, -0.46 for *Stress*, and -0.20 for *Anxiety*.
