# Supplementary material for: Cultivating well-being in engineering graduate students through mindfulness training
Source: PLoS One. 2023 Mar 22;18(3):e0281994. doi: 10.1371/journal.pone.0281994 (PMC10032494; doi:10.1371/journal.pone.0281994)
Supplement: S3 Results — (DOCX) [file pone.0281994.s004.docx]

**S8 Additional Summative Survey Findings**

Phase 1 summative survey: Out of the 35 participants who completed the summative survey, 34 said that they would recommend this training to other graduate students.

Phase 2 summative survey: Out of the 41 participants in Year 2 who responded to the question “Would you recommend this training to other engineering graduate students?”, 40 said that they would recommend this training to other graduate students. Year 1 participants provided slightly more qualified responses with 55 of 59 responding positively, two responding with some reservations, and two responding negatively.

Final summative survey: With a longer time of retrospection, 39 of the 42 individuals who responded to the question “Would you recommend this training to other engineering graduate students?” said yes, while two were neutral and one was negative; a similar rate to the Summative Survey responses (comparisons given in Supplementary Figure S3). Some participants spoke about the longer time needed for them to make connections to the training topics, e.g., “I definitely would recommend this training. Initially it was a challenge for me to adopt these techniques. I actually slipped on performing them for the first semester. Slowly I began to notice the things we discussed in the training in my real life and then it was very easy to adopt the trainings. I might not be using everything we learnt, but I do follow whatever fits my needs. It has been a great help to me. I am happier in my life, and I think it has helped people around me as well.” However, others responded that their use of the training did not persist, e.g., “I have not followed through with the training much beyond the end of the course more than a year ago.”
