# Supplementary material for: Cultivating well-being in engineering graduate students through mindfulness training
Source: PLoS One. 2023 Mar 22;18(3):e0281994. doi: 10.1371/journal.pone.0281994 (PMC10032494; doi:10.1371/journal.pone.0281994)
Supplement: S4 Results — (DOCX) [file pone.0281994.s005.docx]

**S15 Phase 2 Supplementary Results**

***Emotional Style Questionnaire (ESQ)***

*Year 1:* The RMANOVA yielded a significant Time by Group interaction for the overall questionnaire, indicating improved Emotional Health for the intervention group relative to the control group, *F*(1, 87) = 6.68, *p* = 0.011. Paired *t*-tests conducted post hoc revealed a significant increase in emotional health in the intervention group, *t*(37) = -4.32, *p* < 0.001, *d* = 0.55, whereas the change in the control group was not significant, *t*(50) = -1.81, *p* = 0.076, *d* = 0.15. The between-groups effect (*d* = 0.39) was small-to-medium in size. Among the subscales of the ESQ, *Attention*, *F*(1, 87) = 8.39, *p* = 0.005 and *Resilience*, *F*(1, 87) = 4.77, *p* = 0.032 exhibited the largest improvements. The between group *d*’s were 0.46 for *Attention,* and 0.44 for *Resilience.*

*Year 2:* In Year 2, a significant Time by Group interaction for Emotional Health benefiting the intervention group was observed again, *F*(1, 66) = 8.27, *p* = 0.005. Specifically, the intervention group experienced a significant increase in Emotional Health, *t*(34) = -3.17, *p* = 0.003, *d* = 0.46, whereas the control group did not, *t*(32) = 0.47, *p* = 0.641, *d* = -0.05. Among the subscales of the ESQ, *Self-Awareness*, *F*(1, 66) = 11.77, *p* = 0.001 and *Attention*, *F*(1, 66) = 6.33, *p* = 0.014 exhibited the largest improvements. The between group *d*’s were 0.77 for *Self-Awareness*, and 0.44 for *Attention*.

*Years 1 & 2 Combined:* In the combined sample, the Time by Group interaction for Emotional Health was significant, *F*(1, 155) = 14.37, *p* < 0.001. Overall, participants in the intervention group reported higher emotional health over time, *t*(72) = -5.25, *p* < 0.001, *d* = 0.49, whereas no such change was observed in the control group, *t*(83) = -1.15, *p* = 0.254, *d* = 0.07. Significant effects were observed for the *Attention*, *F*(1, 155) = 14.39, *p* < 0.001, *Self-Awareness*, *F*(1, 155) = 12.79, *p* < 0.001, and *Outlook*, *F*(1, 155) = 4.75, *p* = 0.031, subscales. The between group *d*’s were 0.42 for *Attention*, 0.47 for *Self-Awareness,* and *0.26 for Outlook*.

***Ten Item Personality Inventory (TIPI)***

*Year 1:* Among the different facets of the TIPI, we observed a significant Time by Group interaction only for *Neuroticism*, *F*(1, 87) = 4.41, *p* = 0.039. As revealed by post-hoc t-tests, participants in the intervention group perceived themselves as less neurotic over time, *t*(37) = 3.00, *p* = 0.005, *d* = -0.31, whereas no such change occurred in the control group, *t*(50) = 0.35, *p* = 0.729, *d* = -0.03.

*Year 2:* No significant Time by Group interaction was observed for the Neuroticism facet, *F*(1, 66) = 0.79, *p* = 0.379, or any of the other facets.

*Years 1 & 2 Combined:* When both years were combined, the Time by Group interaction for Neuroticism was significant, *F*(1, 155) = 4.88, *p* = 0.029. Specifically, participants in the intervention group perceived themselves as less neurotic over time, *t*(72) = 3.84, *p* < 0.001, *d* = -0.30, whereas no such change occurred in the control group, *t*(83) = 1.25, *p* = 0.214, *d* = -0.08.

No other facet of the TIPI revealed a significant pattern.

***Positive and Negative Affect Schedule (PANAS)***

*Year 1:* The Positive Affect subscale of the PANAS yielded a significant Time by Group interaction, *F*(1, 87) = 15.43, *p* < 0.001. As revealed by post-hoc t-tests, participants in the intervention group reported significantly higher positive affect at Time 2 than Time 1, *t*(37) = -3.75, *p* = 0.001, *d* = 0.43. Those in the waitlist control group on the other hand, showed a marginally significant drop in positive affect, *t*(50) = 1.94, *p* = 0.058, *d* = -0.22.

The interaction for the Negative Affect was also significant, *F*(1, 87) = 5.26, *p* = 0.024. Decomposing this interaction, we found that participants in the intervention group reported experiencing lower Negative Affect at Time 2 relative to Time 1, *t*(37) = 2.55, *p* = 0.015, *d* = -0.30. Negative Affect scores of the participants in the control condition, on the other hand, did not exhibit any change, *t*(50) = -0.87, *p* = 0.391, *d* = 0.10.

*Year 2:* A significant Time by Group interaction for Positive Affect was obtained again, *F*(1, 66) = 9.57, *p* = 0.003. Specifically, the intervention group exhibited significantly improved Positive Affect across time, *t*(34) = -2.99, *p* = 0.005, *d* = 0.48, whereas the waitlist control condition did not report any change, *t*(32) = 1.22, *p* = 0.232, *d* = -0.20.

The interaction for Negative Affect was not significant, *F*(1, 66) = 1.63, *p* = 0.21.

*Years 1 & 2 Combined:* When the samples from both years were combined, both the Positive Affect and Negative Affect subscales of PANAS elicited significant Time by Group interactions, respectively, *F*(1, 155) = 25.41, *p* < 0.001 and *F*(1, 155) = 6.85, *p* = 0.010. Participants in the intervention group reported significantly higher levels of Positive Affect, *t*(72) = -4.60, *p* < 0.001, *d* = 0.45, and significantly lower levels of Negative Affect, *t*(72) = 2.93, *p* = 0.005, *d* = -0.30. Participants in the waitlist control condition, on the other hand, did actually report a significant drop in their Positive Affect, *t*(83) = 2.29, *p* = 0.025, *d* = -0.21, and no change in their Negative Affect, *t*(83) = -0.76, *p* = 0.452, *d* = 0.06.

***Cohen-Hoberman Inventory of Physical Symptoms (CHIPS)***

*Year 1:* The Time by Group interaction for CHIPS was marginally significant, *F*(1, 87) = 3.80, *p* = 0.054. Inspection of effect sizes supported a small-to-medium size reduction in health symptoms favoring the intervention group (between group *d* = -0.38).

*Year 2:* No significant Time by Group interaction was observed for CHIPS, *F*(1, 66) = 1.39, *p* = 0.243.

*Years 1 & 2 Combined:* When the data from both years were combined, the Time by Group interaction reached significance, *F*(1, 155) = 5.06, *p* = 0.026. Paired samples t-tests indicated that participants in the intervention group reported a decreased number of physical health symptoms over time, *t*(72) = 3.01, *p* = 0.004, *d* = -0.37, while the number of symptoms reported by participants in the control condition did not change, *t*(83) = 0.24, *p* = 0.812, *d* = -0.02.

***Mindful Attention and Awareness Scale (MAAS)***

*Year 1:* The Time by Group interaction for the MAAS were significant, *F*(1, 87) = 8.80, *p* = 0.004. Decomposing the interaction, we found that the intervention group reported improved mindful attention and awareness from Time 1 to Time 2, *t*(37) = -2.86, *p* = 0.007, *d* = 0.50, while the waitlist control group did not report any change, *t*(50) = 0.51, *p* = 0.61, *d* = -0. 04.

*Year 2:* A significant Time by Group interaction was obtained again, *F*(1, 66) = 6.89, *p* = 0.011. Paralleling the results from Year 1, the intervention group showed improved mindful attention and awareness, *t*(34) = -2.92, *p* = 0.006, *d* = 0. 66, whereas the waitlist control group did not show such change, *t*(32) = -0.24, *p* = 0.816, *d* = 0. 02.

*Years 1 & 2 Combined:* Combining the data from both years, the RMANOVA results were again significant, *F*(1, 155) = 16.11, *p* < 0.001. In the overall sample, the intervention group exhibited significant gains in mindful attention and awareness, *t*(72) = -4.01, *p* < 0.001, *d* = 0. 58, whereas the control group did not, *t*(83) = -0.29, *p* = 0.773, *d* = -0. 02.

***Five Facet Mindfulness Questionnaire - Short Form (FFMQ-SF)***

*Year 1:* The Time by Group interaction for the overall FFMQ-SF was significant, *F*(1, 87) = 17.02, *p* < 0.001. Specifically, the intervention group reported significantly increased mindfulness over time, *t*(37) = -4.62, *p* < 0.001, *d* = 0.59, whereas the waitlist control group did not show any change, *t*(50) = 0.15, *p* = 0.883, *d* = -0.01. Further analyses revealed that significant effects were observed for the *Act with Awareness*, *F*(1, 87) = 7.50, *p* = 0.007 (between group *d* = 0.48), the *Non-React*, *F*(1, 87) = 6.84, *p* = 0.011 (between group *d* = 0.55) and the *Observe* subscales, *F*(1, 87) = 4.36, *p* = 0.040 (between group *d* = 0.34).

*Year 2:* The analyses yielded a significant Time by Group interaction for the overall FFMQ-SF, *F*(1, 66) = 9.49, *p* = 0.003. As in Year 1, participants in the intervention group reported significantly increased mindfulness from Time 1 to Time 2, *t*(34) = -3.68, *p* = 0.001, *d* = 0.65, whereas the waitlist control group did not show any change, *t*(32) = -0.06, *p* = 0.954, *d* = 0.01. Among the different subscales of the FFMQ-SF, significant effects were observed for the *Observe*, *F*(1, 66) = 6.68, *p* = 0.012 (between group *d* = 0.60), the *Non-React*, *F*(1, 66) = 6.67, *p* = 0.012, (between group *d* = 0.51), and the *Describe*, *F*(1, 66) = 5.24, *p* = 0.025 (between group *d* = 0.52) subscales.

*Years 1 & 2 Combined:* Combining the two years again yielded a significant interaction, *F*(1, 155) = 25.55, *p* < 0.001, pointing to a medium-to-large effect for this variable (between group *d* = 0.62). Overall, participants in the intervention group reported a significant increase in mindfulness from Time 1 to Time 2, *t*(72) = -5.63, *p* < 0.001, *d* = 0.62, whereas no change was reported in the control group, *t*(83) = 0.06, *p* = 0.949, *d* = -0.004. In this larger sample, all the subscales of FFMQ-SF showed significant Time by Group interactions favoring the intervention group, specifically *Observe*, *F*(1, 155) = 11.33, *p* = 0.001 (between group *d* = 0.44), *Describe*, *F*(1, 155) = 8.75, *p* = 0.004 (between group *d* = 0.43), *Act with Awareness*, *F*(1, 155) = 11.53, *p* = 0.001 (between group *d* = 0.47), *Non-Judge*, *F*(1, 155) = 4.52, *p* = 0.035 (between group *d* = 0.28), *Non-React*, *F*(1, 155) = 12.80, *p* < 0.001 (between group *d* = 0.47).

***Research Satisfaction Scale***

*Year 1:* The RMANOVA results for the Research Satisfaction Scale were significant, *F*(1, 85) = 4.36, *p* = 0.040. Specifically, participants in the intervention group reported improved satisfaction with their research from Time 1 to Time 2, *t*(36) = -3.04, *p* = 0.004, *d* = 0.46, whereas the control group did not, *t*(49) = -0.84, *p* = 0.407, *d* = 0.07.

*Year 2:* The Time by Group interaction failed to reach significance, *F*(1, 66) = 2.34, *p* = 0.131.

*Years 1 & 2 Combined:* Analyses conducted in the combined data set yielded a significant Time by Group interaction, *F*(1, 153) = 6.84, *p* = 0.010. Post-hoc paired t-tests again showed that the participants in the intervention group reported improved satisfaction with their research across time, *t*(71) = -4.46, *p* < 0.001, *d* = 0.47, whereas the control group did not show any change, *t*(82) = -1.23, *p* = 0.221, *d* = 0.09.

***Contributive Desire Scale***

*Year 1:* No significant Time by Group interaction was noted for the Contributive Desire Scale, *F*(1, 87) = 0.001, *p* = 0.970.

*Year 2:* The analyses did not reveal a significant Time by Group interaction, *F*(1, 66) = 0.004, *p* = 0.898.

*Years 1 & 2 Combined:* No significant Time by Group interaction was observed for the Contributive Desire Scale in the combined data, *F*(1, 155) = 0.001, *p* = 0.974.

Results for four additional scales are reported in the Additional Supplementary Results for Phase 2: Alternate Uses Task, Remote Associates Test, Pattern Meanings Test, and the Creativity Characteristics Scale
