# Supplementary material for: Cultivating well-being in engineering graduate students through mindfulness training
Source: PLoS One. 2023 Mar 22;18(3):e0281994. doi: 10.1371/journal.pone.0281994 (PMC10032494; doi:10.1371/journal.pone.0281994)
Supplement: S1 References — (DOCX) [file pone.0281994.s006.docx]

**S22 Supplementary References**

Bowden, Edward M, and Mark Jung-Beeman. 2003. 'Normative data for 144 compound remote associate problems', *Behavior Research Methods, Instruments, & Computers*, 35: 634-39.

Cohen, S, and G Williamson. 1988. "Perceived stress in a probability sample of the United States. The social psychology of health: Claremont Symposium on applied social psychology. Edited by: Spacapan S, Oskamp S. 1988." In.: Newbury Park, CA: Sage.

Davidson, Richard J, and Sharon Begley. 2012. *The emotional life of your brain: How its unique patterns affect the way you think, feel, and live--and how you can change them* (Penguin).

Diener, Ed, Derrick Wirtz, William Tov, Chu Kim-Prieto, Dong-won Choi, Shigehiro Oishi, and Robert Biswas-Diener. 2010. 'New well-being measures: Short scales to assess flourishing and positive and negative feelings', *Social Indicators Research*, 97: 143-56.

Doherty, Martin J, and Samantha Mair. 2012. 'Creativity, ambiguous figures, and academic preference', *Perception*, 41: 1262-66.

Guilford, Joy Paul. 1967. 'The nature of human intelligence'.

Henry, Julie D, and John R Crawford. 2005. 'The short‐form version of the Depression Anxiety Stress Scales (DASS‐21): Construct validity and normative data in a large non‐clinical sample', *British Journal of Clinical Psychology*, 44: 227-39.

Mednick, Sarnoff. 1962. 'The associative basis of the creative process', *Psychological Review*, 69: 220.

Silvia, Paul J. 2015. 'Intelligence and creativity are pretty similar after all', *Educational Psychology Review*, 27: 599-606.

Smith, Steven M, and Thomas B Ward. 2012. 'Cognition and the creation of ideas', *The Oxford handbook of thinking and reasoning*: 456-74.

Wallach, Michael A, and Nathan Kogan. 1965. 'Modes of thinking in young children'.
