# Supplementary material for: Cultivating well-being in engineering graduate students through mindfulness training
Source: PLoS One. 2023 Mar 22;18(3):e0281994. doi: 10.1371/journal.pone.0281994 (PMC10032494; doi:10.1371/journal.pone.0281994)
Supplement: S1 Table — Page numbers refer to the relevant sections of The Emotional Life of Your Brain for each week of training (Davidson and Begley 2012). (DOCX) [file pone.0281994.s007.docx]

**S1 Table. Outline of the training curriculum developed by Healthy Minds Innovations.** Page numbers refer to the relevant sections of *The Emotional Life of Your Brain* for each week of training (Davidson and Begley 2012).

| **Weeks 1-2** – **Emotional Style of *Attention***  (pp. 5-8, 67-68, 86-90; pp. 59-65, 225-229, 238-242)  Overview of the six emotional styles and the plasticity of the brain:  Well-being is a trainable skill, a habit to be cultivated  Introduction to *Attention*: science, strategies, challenges, and benefits  Skill building: Mind-on-breath meditations  Cultivating habit: how to build reminders into daily life using cues and triggers |
| --- |
| **Weeks 3-4 - Emotional Style of *Self-Awareness***  (pp. 78-81; pp. 54-56, 234-238)  Introduction to *Self-Awareness:* Science, strategies, challenges, and benefits  Cultivating *Self-Awareness*: body and breath awareness  Skill building: Body-scan meditation  Cultivating habit: how to build mindful habits through reminders, routines, and rewards |
| **Week 5** – **Emotional Style of *Resilience***  (pp. 44-48, 69-72, 242-246)  Introduction to *Resilience*: science, strategies, challenges, and benefits  - the cost of stress  Cultivating *Resilience*: developing insight about how we react to challenges  Skill building: Dealing with challenging situations and Cognitive reframing  Cultivating habit: how to build a daily practice based on the RAIN (recognize, accept, investigate, non-judgment) meditation |
| **Week 6** – **Emotional Style of *Outlook***  (pp. 49-50, 81-85, 229-233)  Introduction to *Outlook*: science, strategies, challenges, and benefits  - Extending resilience: developing an attitude of gratitude  Cultivating a positive *outlook:* Gratitude practice  Skill building: Recognizing potential and Silver-lining meditation  Cultivating habit: how to build a daily practice with a gratitude journal |
| **Week 7** – **Emotional Style of *Social Intuition***  (pp. 51-72, 72-74, 246-249)  Introduction to *Social Intuition*: science, strategies, challenges, and benefits  Cultivating *Social Intuition*: identifying social cues; compassion, empathy, and altruism  Skill building: Connection and Open awareness meditation  Cultivating habit: how to build a daily practice of connecting with others |
| **Week 8** – **Emotional Style of *Sensitivity to Context***  (pp. 57-59, 75-78, 249-252)  Introduction to *Sensitivity to Context*: science, strategies, challenges, and benefits  Cultivating *Sensitivity to Context*: Balanced breath exercise  Skill building: Compassion meditation  Cultivating habit: a review of practices, creating a path forward |
