# Supplementary material for: Cultivating well-being in engineering graduate students through mindfulness training
Source: PLoS One. 2023 Mar 22;18(3):e0281994. doi: 10.1371/journal.pone.0281994 (PMC10032494; doi:10.1371/journal.pone.0281994)
Supplement: S2 Table — (DOCX) [file pone.0281994.s008.docx]

**S3 Table. Intercorrelations between Measures at Pre-Test for Phase 1.**
