# Supplementary material for: Cultivating well-being in engineering graduate students through mindfulness training
Source: PLoS One. 2023 Mar 22;18(3):e0281994. doi: 10.1371/journal.pone.0281994 (PMC10032494; doi:10.1371/journal.pone.0281994)
Supplement: S4 Table — (DOCX) [file pone.0281994.s010.docx]

**S7 Table. Summative Survey Results and Representative Responses for Phase 1 (n = 35).**

| **In what ways has the training impacted your research and other professional work?** | |
| --- | --- |
| Positive: 89%  Neutral: 11%  Negative: 0% | Positive: “I think about mindfulness a lot these days. I use it whenever I am stressed about how much I have left to do, and just focus on the tasks at hand and try to make meaningful progress. I've also used it to think through my research questions more, because I am trying to think of what reviewers and others would object to my reasonings. The mindfulness has helped me envision what others would say in a nonjudgemental [*sic*] way.” |
|  | Positive: “This training has improved my anxiety. It has helped me find ways to ease and control it, which in turn has made me more productive in my research and other professional work.” |
|  | Neutral: "I haven't experienced noticeable changes in my ability to perform research effectively." |
| **In what ways has the training impacted your personal life?** | |
| Positive: 88%  Neutral: 12%  Negative: 0% | Positive: "I think I am slightly less quick to anger and engage in perspective taking." |
|  | Positive: "1. The biggest change is in recognizing that I can separate my emotion and treat it as a temporary state of mind instead of feeling it as a state of being. 2. I'm also more forgiving of my mistakes and of mistakes in others. 3. More focused on listening to people rather than preparing my answer.” |
|  | Neutral: "I don't think the training had much of an impact." |
| **What was most valuable to you about the training?** | |
| Positive:100%  Neutral: 0%  Negative: 0% | Positive: “In a practical sense, being introduced to tools that I can use moving forward. On a personal level, meeting every week and feeling like I was a part of a community of other grad students who were vulnerable and honest about their experiences.” |
|  | Positive: “It was an opportunity to formally practice evidence-based mindfulness methods in the midst of a community.” |
|  | Positive: “The introduction of many different types of mindfulness practices and extremely practical ways to integrate them into daily life was helpful. The encouragement to regularly keep up the practice, supported by research, was also helpful. I had some prior idea of mindfulness practices, but I didn't have an understanding of how to integrate it into daily life, so gaining that was most valuable.” |

| **Would you recommend this training to other engineering graduate students? Why or why not?** | |
| --- | --- |
| Yes: 97%  Maybe: 0%  No: 3% | Yes: “Yes! A lot of people would love better mechanisms to handle their stress but I assume that for many, like me, it takes a formal course for them to spend time learning them.” |
|  | Yes: “Absolutely. So many people internalize their stress and this leads to both mental and physical issues. Academia for graduate students is *extremely* toxic in terms of pressure and the resulting struggles with mental health, and this is a proven way forward through that (at least helping us cope better).” |
|  | Yes: “Most definitely. Graduate work deals with the acceptance of failure and building upon it to improve both professionally as well as personally. This is by no means an easy task, especially in research, and it requires contemplation, the ability to not lose your head but take a step back and analyse [*sic*] things. This is where this training really comes in handy.” |
|  | No: "No, although the resources were useful the classes each week were not very helpful." |
