# Supplementary material for: Cultivating well-being in engineering graduate students through mindfulness training
Source: PLoS One. 2023 Mar 22;18(3):e0281994. doi: 10.1371/journal.pone.0281994 (PMC10032494; doi:10.1371/journal.pone.0281994)
Supplement: S5 Table — (DOCX) [file pone.0281994.s011.docx]

**S9 Table. Summary of participant and completion statistics for Phase 2 - Year 1.**

|  | **University A** | | | **University B** | | | **UA + UB** |
| --- | --- | --- | --- | --- | --- | --- | --- |
|  | exp | control | TOTAL | exp | control | TOTAL | Combined |
| consented and completed pre-test | 31 | 34 | **65** | 26 | 25 | **51** | **116** |
| completed post-test | 23 | 32 | **55** | 16 | 19 | **35** | **90** |
| pre/post-test completion rate | 74% | 94% | **85%** | 62% | 76% | **69%** | **78%** |
| completed summative survey | 16 | 18 | **34** | 14 | 12 | **26** | **60** |
| summative completion rate | 52% | 53% | **52%** | 54% | 48% | **51%** | **52%** |
| completed final summative survey | 14 | 12 | **26** | 11 | 7 | **18** | **44** |
| final summative completion rate | 45% | 35% | **40%** | 42% | 28% | **35%** | **38%** |
