# Supplementary material for: Cultivating well-being in engineering graduate students through mindfulness training
Source: PLoS One. 2023 Mar 22;18(3):e0281994. doi: 10.1371/journal.pone.0281994 (PMC10032494; doi:10.1371/journal.pone.0281994)
Supplement: S6 Table — (DOCX) [file pone.0281994.s012.docx]

**S10 Table. Summary of participant and completion statistics for Phase 2 - Year 2.**

|  | **University A** | | | **University B** | | | **UA + UB** |
| --- | --- | --- | --- | --- | --- | --- | --- |
|  | exp | control | TOTAL | exp | control | TOTAL | Combined |
| consented and completed pre-test | 28 | 23 | **51** | 24 | 20 | **44** | **95** |
| completed post-test | 22 | 21 | **43** | 14 | 12 | **26** | **69** |
| pre/post-test completion rate | 79% | 91% | **84%** | 58% | 60% | **59%** | **73%** |
| completed summative survey | 15 | 10 | **25** | 9 | 9 | **18** | **43** |
| summative completion rate | 54% | 43% | **49%** | 38% | 45% | **41%** | **45%** |
