# Supplementary material for: Cultivating well-being in engineering graduate students through mindfulness training
Source: PLoS One. 2023 Mar 22;18(3):e0281994. doi: 10.1371/journal.pone.0281994 (PMC10032494; doi:10.1371/journal.pone.0281994)
Supplement: S7 Table — ESQ = Emotional Style Questionnaire. TIPI = Ten Item Personality Inventory. PANAS = Positive and Negative Affect Schedule. CHIPS = Cohen-Hoberman Inventory of Physical Symptoms. MAAS = Mindful Attention and Awareness Scale. FFMQ-SF = Five Facet Mindfulness Questionnaire-Short Form. AUT = Alternate Uses Task. For each of the measures, the sample size varied between 37–38 for the intervention group and 50–51 for the control group. * p < 0.05. ** p < 0.01. (DOCX) [file pone.0281994.s013.docx]

**S11 Table. Phase 2 Year 1 Pre- and Post-Test Means (*M*), Standard Deviations (*SD*), Between Group Effect Sizes (*d*), and RMANOVA Results (*F*-value,** *p***-value) for Intervention and Control Groups.** ESQ = Emotional Style Questionnaire. TIPI = Ten Item Personality Inventory. PANAS = Positive and Negative Affect Schedule. CHIPS = Cohen-Hoberman Inventory of Physical Symptoms. MAAS = Mindful Attention and Awareness Scale. FFMQ-SF = Five Facet Mindfulness Questionnaire-Short Form. AUT = Alternate Uses Task. For each of the measures, the sample size varied between 37-38 for the intervention group and 50-51 for the control group. * *p* < 0.05. ** *p* < 0.01.
