# Supplementary material for: Cultivating well-being in engineering graduate students through mindfulness training
Source: PLoS One. 2023 Mar 22;18(3):e0281994. doi: 10.1371/journal.pone.0281994 (PMC10032494; doi:10.1371/journal.pone.0281994)
Supplement: S10 Table — (DOCX) [file pone.0281994.s016.docx]

**S14 Table. Intercorrelations between Measures at Post-Test for Phase 2.**
