# Supplementary material for: Cultivating well-being in engineering graduate students through mindfulness training
Source: PLoS One. 2023 Mar 22;18(3):e0281994. doi: 10.1371/journal.pone.0281994 (PMC10032494; doi:10.1371/journal.pone.0281994)
Supplement: S11 Table — (DOCX) [file pone.0281994.s017.docx]

**S16 Table. Summative Survey Results and Representative Responses for Phase 2 Year 1 (n = 49).**

| **In what ways has the training impacted your research and other professional work?** | |
| --- | --- |
| Positive: 88%  Neutral: 10%  Negative: 2% | Positive: "The mindfulness training has had a very positive impact. I am able to recover from stressful moments much quicker and overall feel more positive towards my research and research community." |
|  | Neutral: "I'm not sure whether it has" |
|  | Negative: "I found the methods un-natural and found it difficult to convince myself to continue practicing mindfulness. This technique possibly can help some people, but my issues are different. ... That being said, I felt that the practices we did helped me calm and fall asleep, even if for short naps. Sleep felt good, but how to resume being alert and hyper-productive afterward? |
| **In what ways has the training impacted your personal life?** | |
| Positive: 91%  Neutral: 9%  Negative: 0% | Positive: "It's encouraged me to pause more and be present, so that I am able to enjoy things more fully. It has also given me more skills to cope with stress." |
|  | Positive: "I feel like I am more resilient to negative events, and better able to catch negative self-talk. I feel more calm and at peace and more grateful for everything I have in life." |
|  | Neutral: "Negligible effects" |
| **What was most valuable to you about the training?** | |
| Positive:100%  Neutral: 0%  Negative: 0% | Positive: "The fact that I periodically remember to take moments to enjoy the present. I don't always have to be looking at my phone or something, I can just be present. Furthermore, I believe some of the mediation [*sic*] practices will be helpful too. I also often do the body scan still before I fall asleep - it helps settle down my mind." |
|  | Positive: "It really gave methods that I could continue to use moving forward through my graduate school career and that I can employ in the heat of the moment to take a step back and calm my thinking." |
|  | Positive: "The chance to meet other engineers who are also interested in meditation, wellness, mental health, and resilience in small group settings. It also improved my emotional intimacy with a lab colleague with whom I was already friendly, which later helped me feel comfortable reaching out to him when I knew he was struggling and also allowed me to ask for help when I had an unexpected health issue. Graduate school is very socially isolating for me especially since I am a single parent and have difficulty going anywhere after business hours." |

| **Would you recommend this training to other engineering graduate students? Why or why not?** | |
| --- | --- |
| Yes: 93%  Maybe: 3%  No: 3% | Yes: "I would recommend the mindfulness training to other graduate students as I think many of the graduate students have similar stressors during the period of their studies and this training has an overall positive effect in our graduate life." |
|  | Maybe: "Yes and no. I thought the content was good and I appreciated all the tools that the course gave me. The book was also valuable. However, I wasn't always impressed with the way time was structured during the in person meetings and am not sure how much I got from those sessions." |
|  | No: "I probably would not, as it was many more weeks of time than I thought necessary to learn the techniques. However, I would probably recommend a shorter workshop for similar skills." |
