# Supplementary material for: Cultivating well-being in engineering graduate students through mindfulness training
Source: PLoS One. 2023 Mar 22;18(3):e0281994. doi: 10.1371/journal.pone.0281994 (PMC10032494; doi:10.1371/journal.pone.0281994)
Supplement: S12 Table — (DOCX) [file pone.0281994.s018.docx]

**S17 Table. Summative Survey Results and Representative Responses for Phase 2 Year 2 (n = 43).**

| **In what ways has the training impacted your research and other professional work?** | |
| --- | --- |
| Positive: 95%  Neutral: 5%  Negative: 0% | Positive: "The mindfulness training helped me to understand my emotional style and how it impacts my work. I believe the mindfulness exercises have also helped me to stay more aware of my emotional state, but I couldn't stick to a meditation routine as I intended. Overall, I think the training helped me to be more productive and avoid burnouts during these difficult pandemic times." |
|  | Positive: "Mindfulness techniques have improved my stress response to formal presentations and public speaking events." |
|  | Neutral: "I love the book immensely, although my practice session was not totally at my comfort level. But I became a meditator afterward…." |
| **In what ways has the training impacted your personal life?** | |
| Positive: 98%  Neutral: 3%  Negative: 0% | Positive: "The training positively impacted my personal life by providing methods to relieve stress." |
|  | Positive: "Have instituted a daily meditation practice which I currently maintain. Has improved communication with my spouse, and provided considerable insight into my mood and thought patterns. I can more easily detect now when something is upsetting to me, and trace the feeling to its source rather than displace the emotion onto other things/people. Acceptance and interest have played a significant role recently in coping with the pandemic." |
|  | Neutral: "It didn't" |
| **What was most valuable to you about the training?** | |
| Positive: 98%  Neutral: 0%  Negative: 2% | Positive: "Introduction to proper meditation techniques. Mindfulness more generally is important, but I find that in order to cultivate it, the daily practice of focused meditation is very important. A large barrier to my previous attempts to institute a daily practice was a lack of confidence in the technique." |
|  | Positive: "I think the most valuable thing to me about the training was increasing my awareness of my own reactions to different things that happen in my day to day life. It has helped me take a second before responding to take inventory of why I am internally reacting a certain way, slow down, and reassess prior to responding or externally reacting." |
|  | Negative: "My time, which is kindda wasted." |
| **Would you recommend this training to other engineering graduate students? Why or why not?** | |
| Yes: 98%  Maybe: 0%  No: 2% | Yes: "Yes I would recommend this to any engineering grad student. It's one of those intangible skills that just makes you a better student and person. Being more relaxed and calm under stressful circumstances definitely benefits anyone who is trying to better themselves and do noteworthy work." |
|  | Yes: "Yes, it changed how I looked at things and helped me to become a better engineer." |
|  | No: "No." |
