# Supplementary material for: Cultivating well-being in engineering graduate students through mindfulness training
Source: PLoS One. 2023 Mar 22;18(3):e0281994. doi: 10.1371/journal.pone.0281994 (PMC10032494; doi:10.1371/journal.pone.0281994)
Supplement: S13 Table — (DOCX) [file pone.0281994.s019.docx]

**S18 Table. Summative Survey Results and Representative Responses for Phase 2 Year 1 Final Summative (n = 44).**

| **In what ways has the training impacted your research and other professional work?** | |
| --- | --- |
| Positive: 75%  Neutral: 25%  Negative: 0% | Positive: "It has made me more aware of my stress triggers/symptoms and helped me cope better. It has also helped me prioritize my mental health over meeting unreasonable deadlines/expectations I sometimes set for myself. I am just as productive but not as stressed or tired." |
|  | Positive: “I appreciated the opportunity to connect with a community of peers about my existing mindfulness practice. I found my office to be more full of friendly faces, and I later volunteered to teach yoga there for an entire semester since I knew there was hunger for mindfulness activities. I also use mindful approaches to negotiate conflict now. I have kept a record of behaviors of collaborators that I have and have not liked, made a note of my feelings about those behaviors, and have made decisions about when and whether to move on from research relationships that were not working. The [*sic*] leaves more room for research relationships that are working.” |
|  | Neutral: "It should have some impact. But now the impact cannot be separated by the influence from the pandemic." |
|  | Neutral: "I couldn't say. I believe it was beneficial, but it's hard to measure benefits of this training vs. other life events." |
| **In what ways has the training impacted your personal life?** | |
| Positive: 88%  Neutral: 10%  Negative: 2% | Positive: "My participation in the training has helped me maintain good mental health which contributes positively in nearly all aspects of my life. I believe that this also helps maintain personal relationships with others, as well as a general sense of well-being." |
|  | Neutral: "No impact. Haven’t been able to establish lasting habits." |
|  | Negative: "I haven't noticed a significant change. During the time when I was practicing the methods learned, I found that I was occasionally more stressed." |

| **What was most valuable to you about the training?** | |
| --- | --- |
| Positive: 90%  Neutral: 8%  Negative: 3% | Positive: "The normalization of meditation, stress, and caring for mental and physical well being. Developing tools and practicing them. Being with other graduate students and realizing that we struggle with similar things." |
|  | Neutral: "not sure" |
|  | Negative: "The awareness of how distracted my mind is, and how I have less control over thoughts occupying my mind than I thought. I haven't been successful in alleviating the mental distractions." |
| **Would you recommend this training to other engineering graduate students? Why or why not?** | |
| Yes: 93%  Maybe: 5%  No: 3% | Yes: "Yes - it is a valuable tool in your graduate student toolbox. While graduate students have lots of resources for improving their technical or research skills, we come across fewer opportunities to improve our self-awareness and ability to navigate anxiety/stress. This training was an easy time commitment and a great crash course in mindfulness for even the busiest graduate student!" |
|  | Maybe: "Maybe. Some could get things out of it. Some might not. If they are discontent and looking for ways to change things up in their life I would suggest it. If people are already content and seem to have things figured out in their own way I probably wouldn't." |
|  | N0: "I would not recommend this to other graduate students. While the information was useful and overall great, I feel that the number of classes should have been condensed. I felt like it was clear there wasn't enough content to fill each class and I would have preferred have less courses with more information." |
