# Supplementary material for: Cultivating well-being in engineering graduate students through mindfulness training
Source: PLoS One. 2023 Mar 22;18(3):e0281994. doi: 10.1371/journal.pone.0281994 (PMC10032494; doi:10.1371/journal.pone.0281994)
Supplement: S1 Fig — Summative Data Comparison Across Years for Positive, Negative and Neutral Responses to “In what ways has the training impacted your research and other professional work?”. (DOCX) [file pone.0281994.s020.docx]

**S19 Figure. Impact on Research.** Summative Data Comparison Across Years for Positive, Negative and Neutral Responses to “In what ways has the training impacted your research and other professional work?”
