# Supplementary material for: Cultivating well-being in engineering graduate students through mindfulness training
Source: PLoS One. 2023 Mar 22;18(3):e0281994. doi: 10.1371/journal.pone.0281994 (PMC10032494; doi:10.1371/journal.pone.0281994)
Supplement: S3 Fig — Summative Data Comparison across Years for Yes, Maybe, and No Responses to “Would you recommend this training to other engineering graduate students?”. (DOCX) [file pone.0281994.s022.docx]

**S21 Figure. Recommend Training to Others.** Summative Data Comparison across Years for Yes, Maybe, and No Responses to “Would you recommend this training to other engineering graduate students?”
